# A pore locus in the E1 domain differentially regulates Cx26 and Cx30 hemichannel function

**DOI:** 10.1085/jgp.202313502

**Published:** 2024-09-20

**Authors:** Helmuth A. Sanchez, Lina Kraujaliene, Vytas K. Verselis

**Affiliations:** 1 Centro Interdisciplinario de Neurociencia de Valparaíso, Facultad de Ciencias, Universidad de Valparaíso, Valparaíso, Chile; 2 Institute of Cardiology, Lithuanian University of Health Sciences, Kaunas, Lithuania; 3 Dominick P. Purpura Department of Neuroscience, Albert Einstein College of Medicine, Bronx, NY, USA

## Abstract

Connexins (Cxs) function as gap junction (GJ) channels and hemichannels that mediate intercellular and transmembrane signaling, respectively. Here, we investigated the proximal segment of the first extracellular loop, E1, of two closely related Cxs, Cx26 and Cx30, that share widespread expression in the cochlea. Computational studies of Cx26 proposed that this segment of E1 contains a parahelix and functions in gating. The sequence of the parahelix is identical between Cx26 and Cx30 except for an Ala/Glu difference at position 49. We show through cysteine-scanning and mutational analyses that position 49 is pore-lining and interacts with the adjacent Asp50 residue to impact hemichannel functionality. When both positions 49 and 50 are charged, as occurs naturally in Cx30, the hemichannel function is dampened. Co-expression of Cx30 with Cx26(D50N), the most common mutation associated with keratitis-ichthyosis-deafness syndrome, results in robust hemichannel currents indicating that position 49–50 interactions are relevant in heteromerically assembled hemichannels. Cysteine substitution at position 49 in either Cx26 or Cx30 results in tonic inhibition of hemichannels, both through disulfide formation and high-affinity metal coordination, suggestive of a flexible region of the pore that can narrow substantially. These effects are absent in GJ channels, which exhibit wild-type functionality. Examination of postnatal cochlear explants suggests that Cx30 expression is associated with reduced propagation of Ca^2+^ waves. Overall, these data identify a pore locus in E1 of Cx26 and Cx30 that impacts hemichannel functionality and provide new considerations for understanding the roles of these connexins in cochlear function.

## Teaser

Two connexins, Cx26 and Cx30, are expressed in the cochlea and play vital roles in hearing. Sanchez et al. identified a sequence difference in their E1 domains that differentially affects their abilities to operate as hemichannnels and function in transmembrane signaling.

## Introduction

Pathologies affecting hearing and skin represent one of the strongest links between connexin (Cx) channel dysfunction and human disease (Srinivas et al., 2018). For hearing loss, mutations in the *GJB2* gene encoding Cx26, one of 21 distinct Cx genes in humans, account for as much as 50% of severe-to-profound inherited deafness cases across diverse ethnic populations (Angeli et al., 2012; Apps et al., 2007; Chan et al., 2010; Duman and Tekin, 2012). The basis for Cx-mediated cochlear function and pathogenesis must take into account two fundamental considerations, the widespread expression of two connexins, Cx26 and Cx30, and the abilities of these Cxs to function as intercellular gap junction (GJ) channels and as undocked hemichannels, each of which serve different cellular functions.

Connexins assemble as hexameric connexons or hemichannels, which when trafficked to the plasma membranes can dock with hemichannels in apposing-cell membranes to form GJ channels. The GJ channel configuration serves as a direct communication pathway between cells with a pore size large enough to permit transmission of chemical signals and metabolites. Hemichannels that remain undocked also have the capacity to function in the plasma membrane, thereby serving as mediators of transmembrane and paracrine signaling. Molecules proposed to enter/exit cells through Cx hemichannels include, but are not limited to, ATP, glutamate, IP_3_, Ca^2+^, glutathione, NAD^+^, and cyclic nucleotides (Cheung et al., 2022; Evans et al., 2006; Leybaert et al., 2003; Siller-Jackson et al., 2008; Tong et al., 2015).

In the cochlea, Cx26 and Cx30 are extensively co-expressed in various supporting cells of the organ of Corti and in the cells that make up the lateral wall (Ahmad et al., 2003; Forge et al., 2003; Lautermann et al., 1998; Liu et al., 2017; Liu and Zhao, 2008; Zhao and Yu, 2006). Their expression patterns overlap substantially, but not completely so that cochlear GJ channels and hemichannels could consist of homomers and mixed heteromers of Cx26 and Cx30, making for a limited, but still complex set of functional possibilities. In principle, dysfunction of GJ channels and/or hemichannels associated with either Cx26 or Cx30 can contribute to disease pathogenesis. As it turns out, mutations in Cx26 account for the vast majority of Cx-linked congenital deafness cases in humans. Most of these mutations are recessive and result in non-syndromic deafness. Truncations, deletions, or frameshifts are common non-syndromic mutants and, thus, produce a loss of both GJ- and hemichannel-mediated functions. Although less common, Cx26-mediated deafness can be syndromic, with hearing loss accompanied by skin disorders. These mutations are typically missense and dominant in nature and are ascribed to one of several syndromes depending on the phenotypes exhibited by patients (Lee and White, 2009; Srinivas et al., 2018; Xu and Nicholson, 2013). Keratitis-ichthyosis-deafness (KID) syndrome is a particularly severe disorder that can result in serious disability and even mortality due to infectious or neoplastic cutaneous complications (Coggshall et al., 2013). KID mutations can result in loss of GJ channel function, but functioning hemichannels exhibiting aberrant properties is a common property. These findings, together with a lack of a skin phenotype associated with loss of Cx26 suggest hemichannel gain of function may be a significant or determining factor in the pathogenesis of Cx-mediated syndromic deafness (Sanchez and Verselis, 2014; Verselis, 2019).

Mouse models have shown that while genetic deletion of Cx26 invariably results in deafness, deletion of Cx30 does not as long as sufficient expression levels of Cx26 are maintained; expression of Cx26 and Cx30 are coregulated through the sharing of cis-acting elements between their encoding regions that lie within the DFNB1 locus (Ahmad et al., 2007; Boulay et al., 2013; Moisan et al., 2019). The reciprocal experiment of maintaining higher Cx30 expression in Cx26 null mice did not prevent hearing loss (Qu et al., 2012). Thus, both human disease and animal models point to Cx26 as particularly crucial for hearing, suggesting that functional differences between Cx26 and Cx30 could be a contributing factor to their differential impacts on cochlear function.

Structure-function studies indicate that the N-terminal half of a Cx protein contains the core structural elements that define channel gating, conductance, and permeability (Hu and Dahl, 1999; Kronengold et al., 2012; Trexler et al., 2000; Verselis, 2009; Verselis et al., 1994). Within this core, residues located in the N-terminal domain, NT, and the first extracellular loop domain, E1, near the border with the first transmembrane domain, TM1, have been shown to form the bulk of the pore (Kronengold et al., 2003; Oh et al., 2008; Zhou et al., 1997) and to participate in voltage-dependent gating (Tang et al., 2009; Verselis et al., 2009). The first crystal structure of a Cx was that of the Cx26 GJ channel and confirmed that E1 and NT domains are core contributors to the pore (Maeda et al., 2009). Subsequent computational studies yielded a refined structure that was proposed to more closely represent an open Cx26 hemichannel configuration (Kwon et al., 2011). In this refined structure, a segment of the Cx26 pore in E1, between residues E42 and F51, adopted an imperfect 3_10_ helix (a parahelix) based on a modified pattern of backbone hydrogen bonding. All-atomic molecular dynamic simulations suggested that the structural stability of the parahelix is governed by interacting electrostatic and van der Waals networks that upon rearrangement can narrow the pore lumen in the region of the parahelix to ≤4 Å and, thus, act to restrict ionic conduction (Bargiello et al., 2018; Kwon et al., 2011, 2012). Mutations at two positions in Cx26 located within this parahelical region of E1, G45E, and D50N/A are associated with KID syndrome, and these mutant Cx26 channels were shown to exhibit a multiplicity of aberrant properties (Gerido et al., 2007; Mhaske et al., 2013; Sánchez et al., 2010; Sanchez et al., 2013; Stong et al., 2006).

The sequence of the parahelix is identical between Cx26 and Cx30 except for position 49, which is an Ala in Cx26 and a Glu in Cx30. Here, we show that this A/E49 is pore-lining in functionally active Cx26 and Cx30 hemichannels and that the Ala/Glu difference differentially impacts GJ and hemichannel function. Effects on hemichannel function involve an interaction between modifications and or substitutions introduced at position 49 and the adjacent pore-lining position, D50. Both A49C and E49C substitutions produced tonic inhibition of Cx26 and Cx30 hemichannels, respectively, through disulfide formation and high-affinity metal coordination, suggestive of a flexible parahelical region that plausibly participates in gating. GJ channels were refractory to the effects Cys-substitution, suggesting that docking may alter interactions among residues involving the parahelix. Overall, our findings are relevant for understanding the functional roles of GJ channels and hemichannels in cochlear physiology as well as disease pathogenesis given that D50N is the most common KID syndromic deafness mutation in Cx26 (Mazereeuw-Hautier et al., 2007).

## Materials and methods

### Construction of Cx26 and Cx30 mutants

Human wild-type (WT) Cx26 and Cx30 were cloned into the BamHI restriction site of the pCS2^+^ expression vector for functional studies and exogenous expression. To attach the monomeric fluorescent proteins msfGFP and mScarlet to the C-termini of Cx26 and Cx30, respectively, the nucleotide sequence of msfGFP or mScarlet was fused in the frame via a seven amino acid linker (5′-ACG​CGT​ACG​CGG​CCG​CTC​GAG-3′). Cx-fusion proteins and site-directed mutations were constructed by GenScript. All constructs were verified by sequencing.

### Exogenous expression of connexins in *Xenopus* oocytes

*Xenopus laevis* oocytes were purchased from Xenopus I Corp. The oocytes were defolliculated by incubating them for 2 h at room temperature in a solution containing 30 mg collagenase B from *Clostridium histolyticum* (MilliporeSigma) dissolved in 15 ml of a solution containing (in mM) 100 NaCl, 2 KCl, 1 MgCl_2_, 10 glucose, and 10 HEPES, pH adjusted to 7.6. Defolliculated oocytes were placed in a modified ND96 solution containing (in mM) 100 NaCl, 2 KCl, 1 MgCl_2_, 1.8 CaCl_2_, 10 glucose, 10 HEPES, and 5 pyruvate, pH adjusted to 7.6 to and allowed to recover overnight at 16−18°C.

Oocytes were coinjected with 50 nl of an aqueous solution containing ∼1 pLg/Il RNA and 0.25 µg/Ipl of and antisense oligonucleotide 5′-GCY​TTA​GTA​ATT​CCC​ATC​CTG​CCA​TGT​TTC-3′ that is complementary to endogenous *Xenopus* Cx38 commencing at nt −5 (Ebihara et al., 1989). mRNA was prepared from linearized plasmid DNA with mMessage mMachine SP6 RNA kits (Invitrogen) according to the manufacturer’s protocol. The mRNA was purified using QIAquick PCR purification columns (QIAGEN Sciences, Inc.). mRNA bound to the column was eluted with 30–40 μl of an aqueous solution of 8 pmol/ml DNA antisense to the endogenous *Xen*Cx38 (Trexler et al., 2000). RNA concentrations were measured using a NanoDrop 2000 Spectrophotometer (Thermo Fisher Scientific), and tubes containing equal concentrations were prepared by adding appropriate amounts of nuclease-free water. Injections were accomplished using a Nanoject II auto-nanoliter injector (Drummond Scientific Company). Injected oocytes were maintained at 16–18°C in modified ND96. Oocyte cell pairs were obtained by manually devitellinizing individual oocytes in a hypertonic solution containing (in mM) 200 K-aspartate, 20 KCl, 1 MgCl_2_, and 10 mM HEPES, pH 7.6. Following devittelinization, oocytes were paired in standard 15-mm tissue culture dishes coated with 1% agarose to prevent mechanical damage.

### Reagents

Dithiothreitol (DTT), N,N,N′,N′-tetrakis(2-pyridylmethyl)ethylenediamine (TPEN) were purchased from Millipore Sigma. Methanethiosulfonate (MTS) reagents, 2-aminoethyl methanethiosulfonate (MTSEA), 2-trimethylammonioethylmethane thiosulfonate (MTSET), and 2-sulfonatoethylmethane thiosulfonate (MTSES) were purchased from Anatrace.

For MTS reagents, aliquots of dry powder were prepared and stored in microcentrifuge tubes at −20°C. Prior to each experiment, aliquots of MTSET and MTSES were dissolved in distilled water to stock concentrations of 100–200 mM and chilled on ice. Dilutions were made into appropriate perfusion solutions just prior to application to a final concentration of 0.2 mM for MTSET and 2 mM for MTSES. The activity of MTS reagents was periodically checked using the TNB assay (Karlin and Akabas, 1998). DTT and TPEN were prepared daily from frozen stocks.

### Electrophysiological recordings

To measure macroscopic hemichannel currents, *Xenopus* oocytes were placed in a polycarbonate RC-1Z recording chamber (Warner Instruments) with inflow and outflow compartments. The outflow compartment contained agar bridges connected to a VG-2A virtual ground bath clamp (Molecular Devices). Solutions were applied through reservoirs controlled electronically by pinch values (Warner Instruments). All recordings were obtained with a GeneClamp 500 two-electrode voltage clamp amplifier (Molecular Devices). Both current-passing and voltage-recording pipettes contained 1 M KCl. During recording, oocytes were perfused with simple salt solutions that contained (in mM) 100 NaCl, 1 MgCl_2_, and 10 HEPES to which Ca^2+^ was added to achieve desired values. pH was adjusted to 7.8 using NaOH. Steady-state currents were measured at −40 mV. Steady-state conductance–voltage (G-V) relationships were obtained from voltage ramps applied from +40 to −100 mV, 600 s in duration. Cells were held at a holding potential of −20 mV. Prior to initiating the ramps, oocytes were stepped to +40 mV for 30 s to allow conductance to reach steady state. Currents were filtered at 200 Hz and digitized at 1–2 kHz.

For measurements of GJ conductance (g_j_), each oocyte of a pair was clamped independently using a two-electrode voltage clamp to a common potential. Transjunctional voltages, V_j_s, were applied by stepping the voltage in one cell while keeping the voltage in the other cell constant. Junctional current (I_j_) was measured as the current change in the unstepped cell. To obtain g_j_-V_j_ relationships, voltage steps 10 s in duration were applied over a range of ±120 mV in 10 mV increments in one cell; the voltage in the other cell remained constant. Each voltage step was preceded by a small, brief prepulse of constant amplitude so that a family of currents could be normalized if the expression level changed over the course of an experiment. The cells were allowed to recover for 60 s between voltage steps. Currents were filtered at 200 Hz and digitized at 1–2 kHz. Steady-state g_j_–V_j_ relationships were obtained by extrapolating exponential fits of the data from voltage steps to *t* = ∞ as previously described (Verselis et al., 1994). Only cell pairs with g_j_ values ≤10 µS were used in generating g_j_–V_j_ relationships to avoid the effects of series access resistance on voltage dependence (Wilders and Jongsma, 1992).

Data were acquired with AT-MIO-16X D/A boards from National Instruments using custom acquisition and analysis software (written by E. Brady Trexler, Gotham Scientific, Hasbrouck Heights, NJ).

### Cochlear explant cultures

The organ of Corti was isolated from C57/BalbC mice at postnatal day 4 or 7 following the protocol of Parker et al. (2010). Briefly, following the removal of the temporal bones, the bony labyrinth of the cochlea was removed to expose the spiral ligament and organ of Corti. The spiral ligament was secured to the dish allowing separation of the organ of Corti starting from the basal end. The extracted organ of Corti was plated on glass coverslips coated with polyornithine: laminin in a 1:1 ratio and supplemented with 20% fetal bovine serum (FBS). The dissected organ was then cultured in Dulbecco’s modified Eagle medium (DMEM) containing 5% FBS, 5% horse serum, and 1 µg/ml ampicillin and maintained for no >2 days in vitro.

### Antibody staining

Primary antibodies used were directed against myosin-7a (PA1-936; Life Technologies), Cx26 (335800 and 710500; Life Technologies), and Cx30 (712200; Life Technologies). Secondary antibodies used were goat anti-rabbit (A11034, and A11036; Life Technologies) and goat anti-mouse (A11029; Life Technologies). Briefly, cochlear explants were fixed with paraformaldehyde (4% dissolved in PBS) for 20 min at room temperature (RT), rinsed with PBS, and stored at 4°C. Explants were permeabilized with ethanol (70%, −20°C for 20 min), followed by incubation with ammonium chloride (50 mM in PBS, 30 min at room temperature [RT]). Next, cultures were preincubated with blocking solution (20% normal goat serum and 5% BSA dissolved in PBS) for 1 h at RT, followed by overnight incubation with primary antibodies (4°C). The next day, primary antibodies were washed out with PBS, and secondary antibodies were applied in a blocking solution for 1 h at RT. Finally, after washing out secondary antibodies with PBS and a final rinse in distilled water, explants were mounted (Fluoromount-G, EMS) and stored at 4°C until the next day for visualization on an Olympus BX-51W1 microscope equipped with 20× UMPlanFL N, 0.5 N.A., 40× LUMPlanFL N, 0.8 N.A. and 60× LUMPlanFL/IR 0.9 N.A. objectives, a Hamamatsu ORCA flash 4.0 camera (Hamamatsu Photonics), a Sutter DG4 light source (Sutter Instruments), appropriate filters, 39002 for Alexa Fluor 488, 49008 for AlexaFluor 568 (Chroma Technology), and imaging software, MetaFluor ver 7.8.13, 64-bit (Molecular Devices). All images were acquired at room temperature.

### Calcium imaging in cochlear explants

The monitoring of intracellular Ca^2+^ was performed following previous protocols (Sánchez et al., 2009). Briefly, Fura-2 AM stocks (50 mM in DMSO) were mixed with Pluronic acid (mix 1:1) and dissolved in DMEM media without serum (10 µM final concentration) and applied to cochlear explants for 30 min at 37°C. The cochlear explants were rinsed and bathed in an extracellular solution adapted for cochlea explant cultures (Beltramello et al., 2005). Ca^2+^ imaging was performed on an Olympus BX-51W1 microscope using a Lambda DG-4, (Sutter Instruments, Inc.) illumination system that allowed rapid switching between 340 and 380 nm excitation wavelengths. Emission was monitored at 510 nm. Images were acquired using MetaFluor software, ver 7.8.13, 64-bit (Molecular Devices), and an ORCA-Flash 4.0 digital CMOS camera (Hamamatsu Photonics) equipped with a Camera-Link to allow rapid, full-frame imaging. To induce Ca^2+^ waves, ATP was dissolved in bath solution (40 µM) and loaded in borosilicate pipettes pulled to ∼5 MΩ resistance. A brief pressure pulse was applied to a pipette positioned just above a chosen region within the cochlear explant.

For image analyses, a sequence detailing Ca^2+^ activity was summed into the 16-bit image to establish a weighted colormap displaying ratiometric output across time. The red channel was split off from the ratiometric output obtained in MetaFluor to isolate the intensities reflecting the Ca^2+^ signal. Signal intensity was thresholded in MetaMorph (Molecular Devices) to establish an inclusive binary mask for Ca^2+^ activity. Via a logical AND operation, the mask was used to sum integrated intensities of three sequential frame pairs into a 16-bit image to display the Ca^2+^ activity. To remove hot pixels and background noise, binary operations were performed (“Remove single pixels,” then “Erode” process [4 neighbor] with a final “Remove single pixels” pass). The Ca^2+^ activity colormap was overlaid over a cropped and summed projection of the tissue to reflect cell strata.

### Statistical analysis

For group comparisons, we applied either one-way ANOVA with post hoc Tukey’s multiple comparison test. Paired and unpaired t-tests were applied for comparisons of the same samples under two conditions, e.g., high and low Ca^2+^. Results are presented in the figure legends. Analyses were performed using GraphPad Prism v10.

### Online supplemental material

**Figure S1.**
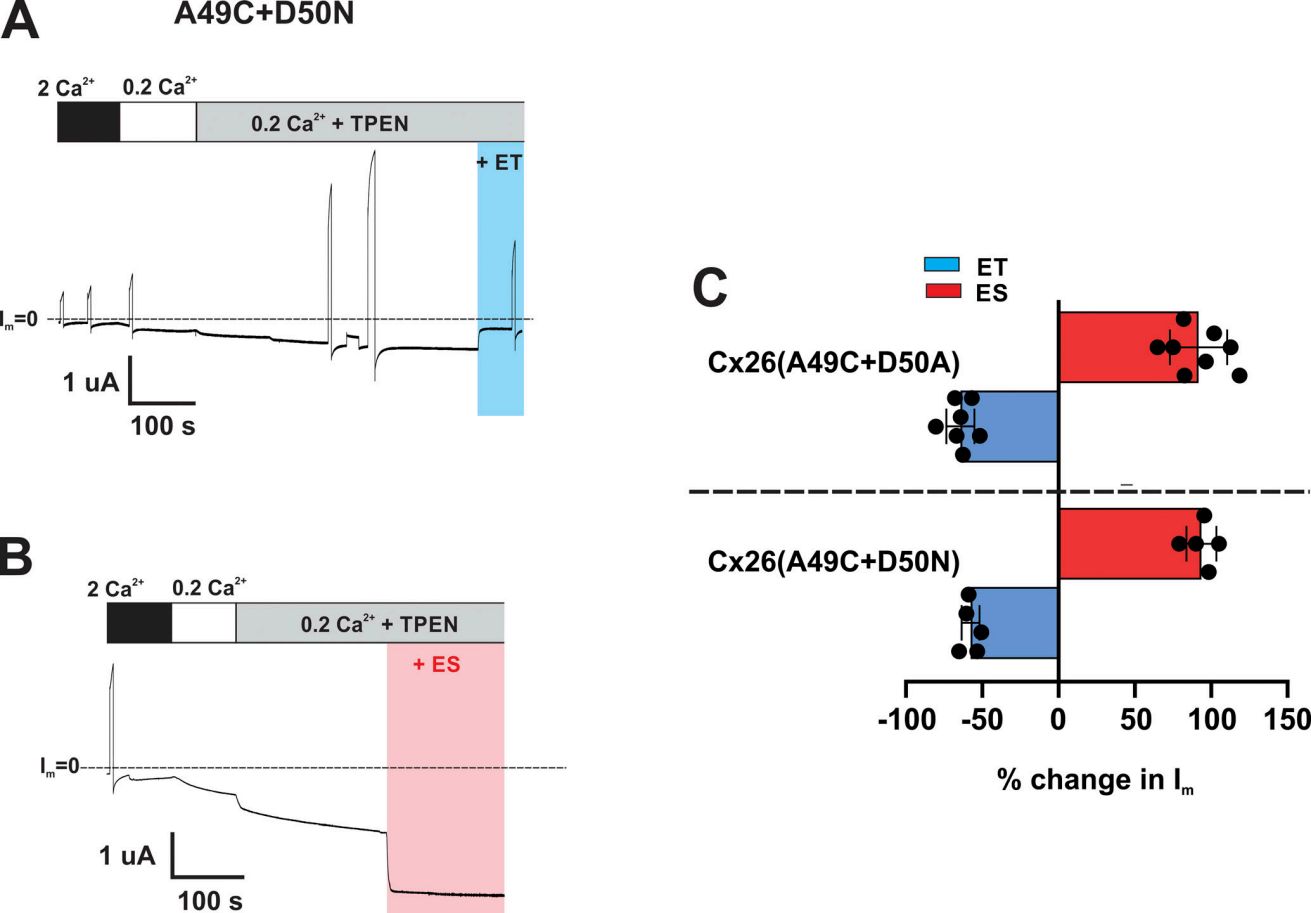
**Neutralization of D50 with and Ala or Gln in A49C hemichannels produces similar effects on MTS modifications. (A and B)** Recordings of hemichannel currents from *Xenopus* oocytes expressing the double mutant Cx26(A49C+D50N). Cells were voltage clamped at −40 mV and currents were evident in 0.2 Ca^2+^ without DTT. Application of TPEN showed modest potentiation consistent with some retained sensitivity to metals. MTS applications produced robust effects that were opposite in sign for MTSET and MTSES; MTSET (0.2 mM) produced a rapid and sustained decrease in current (blue shaded area) and MTSES (2 mM) a rapid and sustained increase in current (red shaded area). **(C)** Summary of MTS effects comparing A49C+D50A and A49C+D50N hemichannels. Filled circles represent individual data points obtained in different oocytes. Bars represent mean % changes in current (I_m_) following MTS application. Cx26(A49C+D50A) + ET (*n* = 7); Cx26(A49C+D50A) + ES (*n* = 8); Cx26(A49C+D50N) + ET (*n* = 5); Cx26(A49C+D50N) + ES (*n* = 5).

**Figure S2.**
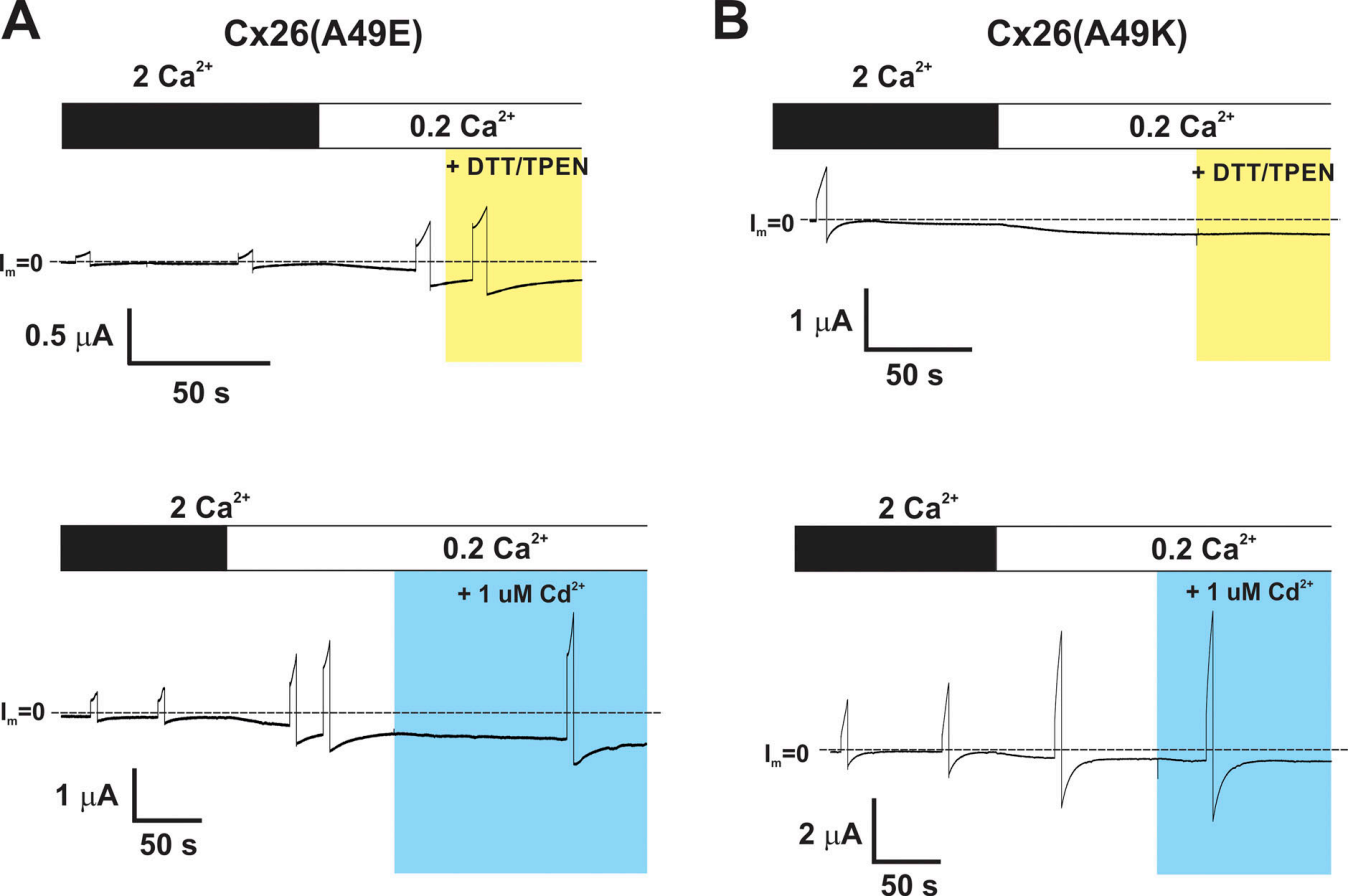
**A49E and A49K hemichannels are insensitive to DTT/TPEN and 1 uM Cd**^**2+**^**. (A and B)** Recordings of hemichannel currents from *Xenopus* oocytes expressing Cx26(A49E) (A) and Cx26(A49K) (B). Cells were voltage clamped at −40 mV and currents. Exposure to 0.2 Ca^2+^ led activated both A49E and A49K currents. Application of a combination of DTT (200 μM) +TPEN (10 μM) or 1 μM Cd^2+^ showed no effects in contrast to A49C hemichannels.

**Figure S3.**
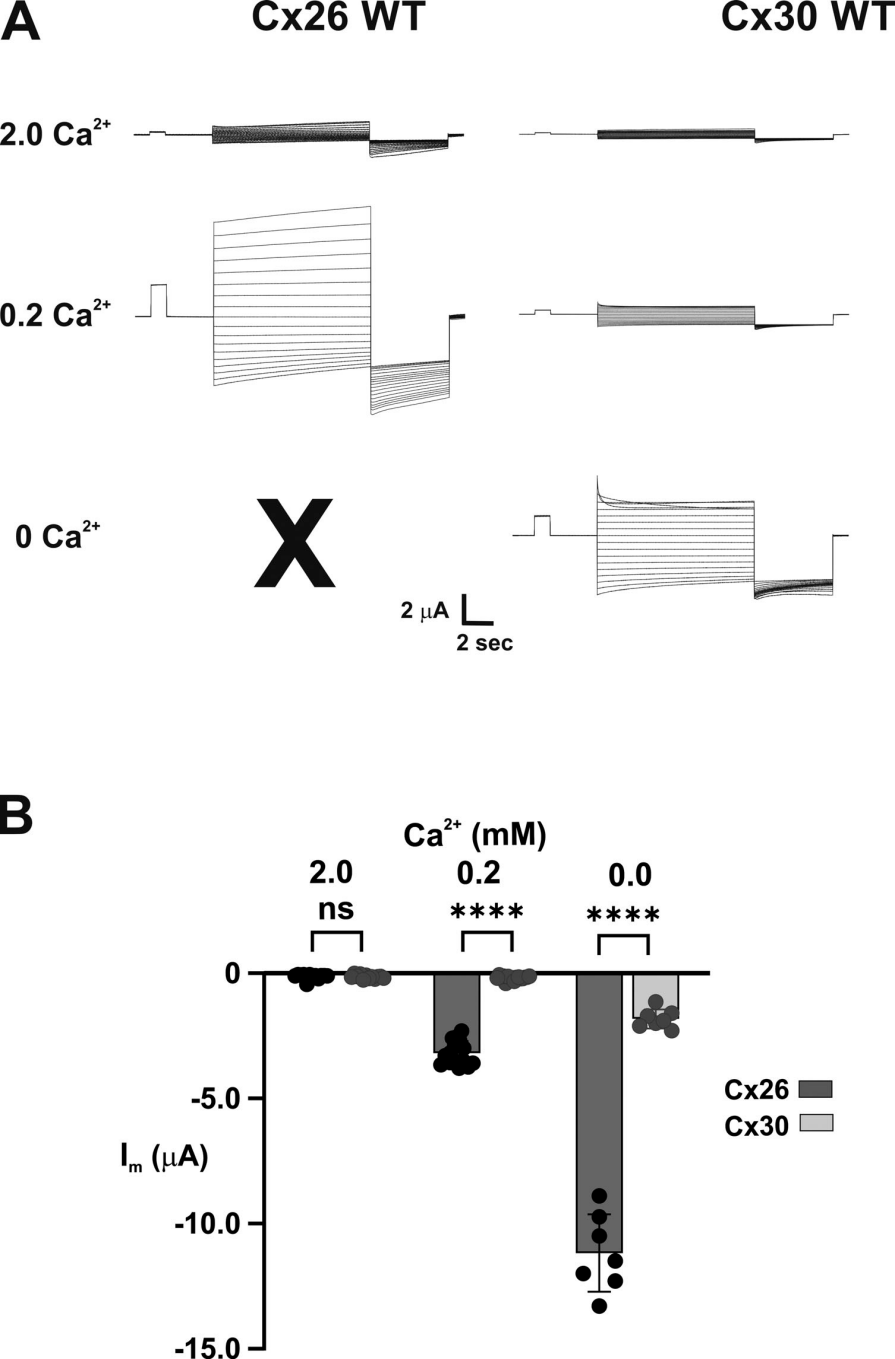
**Cx26 and Cx32 hemichannels show differential levels of activation with changes in extracellular Ca**^**2+**^**. (A)** Examples of families of currents for Cx26 and Cx30 hemichannels in 2, 0.2 and 0 mM (added) extracellular Ca^2+^. Each series represents currents from the same cell. Cx26 currents increased markedly from 2 to 0.2 mM Ca^2+^, whereas Cx30 currents remained small. In 0 mM added (nominal) Ca^2+^, Cx26 currents were excessively large such that cells could not be reliably held in voltage clamp over the entire voltage range necessary to obtain a comparable family of currents. Cx30 currents, however, could be recorded and increased substantially in 0 mM added Ca^2+^. The voltage protocol consisted of a brief prepulse step from a holding potential of −20 to +10 mV followed by 10-s steps ranging from +60 to −100 followed by step to −120 mV. **(B)** Summary of experiments from different cells showing mean current levels evaluated at −40 mM at each Ca^2+^ concentration. At this voltage, both Cx26 and Cx30 hemichannels showed little or no detectable currents from baseline in 2 mM Ca^2+^. Both Cx26 and Cx30 hemichannels showed significant activation when Ca^2+^ was lowered with Cx26 hemichannels exhibiting substantially larger currents than Cx30 hemichannels. Bars represent mean ± SD. Significance among groups was tested using a one-way ANOVA and post-hoc Tukey’s adjusted for pairwise comparison. Asterisks denote statistical significance (****P value <0.0001; ns denotes no significance). P value for Cx26 and Cx30 in 2 mM Ca^2+^ (0.9999). Cx26 2 Ca^2+^ (*n* = 12), 0.2 Ca^2+^ (*n* = 15), 0 Ca^2+^ (*n* = 7); Cx30 2 Ca^2+^ (*n* = 12), 0.2 Ca^2+^ (*n* = 16), 0 Ca^2+^ (*n* = 7).

**Figure S4.**
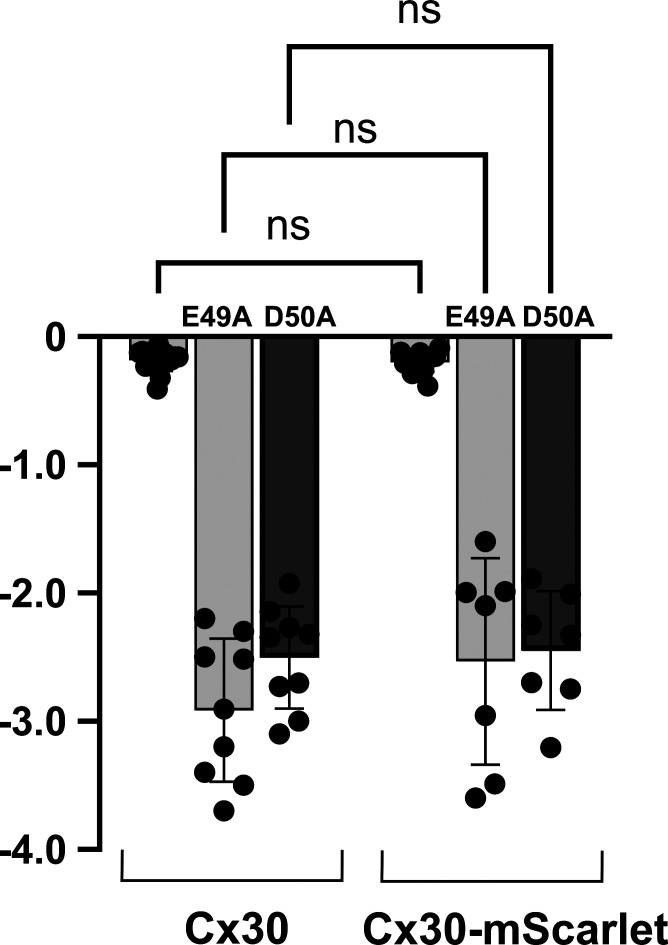
**Cx30 hemichannels with mScarlet attached to its C-terminus behave similar to WT Cx30 hemichannels.** Summary of data comparing Cx30 and Cx30mScarlet currents expressed in *Xenopus* oocytes. Bar graphs showing mean values of hemichannel currents measured at a holding potential of −40 mV in solutions containing 0.2 mM Ca^2+^. Filled circles represent individual measurements in different oocytes. Both Cx30 and Cx30-mScarlet hemichannels exhibited small currents marginally detectable above baseline. E49A and D50A in Cx30-mScarlet hemichannel led to robust currents, consistent with the effects observed in Cx30 hemichannels. Oocytes were injected with the same concentrations of cRNA and measurements were obtained between 24 and 48 h after injection. Bars represent mean ± SD. Significance among selected groups was tested using a one-way ANOVA and post-hoc Tukey’s adjusted for pairwise comparison. ns denotes no significance. There were no significant differences among indicated pairs; P values for WT Cx30 versus Cx30-mScarlet (0.9994), Cx30(E49A) versus Cx30(E49A)-mScarlet (0.1738), and Cx30(D50A) versus Cx30(D50A)-mScarlet (0.9894). Cx30 (*n* = 16), Cx30-mScarlet (*n* = 16), Cx30(E49A) (*n* = 9), Cx30(E49A)-mScarlet (*n* = 7), Cx30(D50A) (*n* = 9), Cx30(D50A)-mScarlet (*n* = 7).

**Figure S5.**
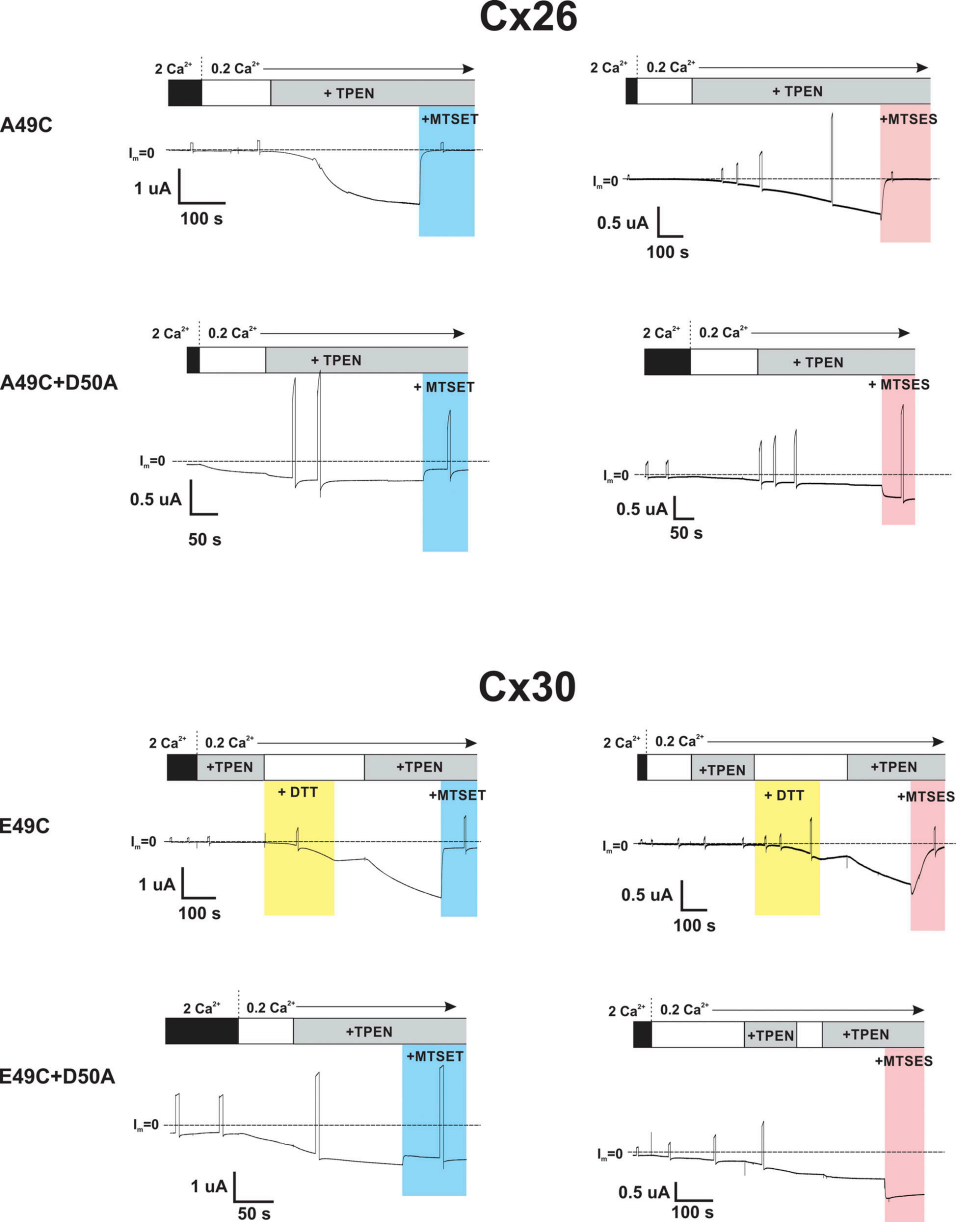
**Effects of MTS reagents on Cx26 and Cx30 hemichannel currents with Cys substitution at position 49 alone and in combination with the D50A substitution.** Shown are recordings of hemichannel currents in *Xenopus* oocytes voltage clamped at −40 mV. Cx26(A49C) hemichannels were previously exposed to DTT. For Cx30, the effects of DTT and TPEN are illustrated. In each case, following application of 10 μM TPEN in low (0.2 mM Ca^2+^) MTS reagents were applied in the continued presence of TPEN. The examples illustrate the similar effects of MTS application in both Cx26 and Cx30 hemichannels carrying a Cys substitution at position 49 and the dramatic effects of neutralization of D50 with a D50A substitution. Note that MTSES reverses in sign with D50 neutralized, showing a sustained increase in current.

**Figure S6.**
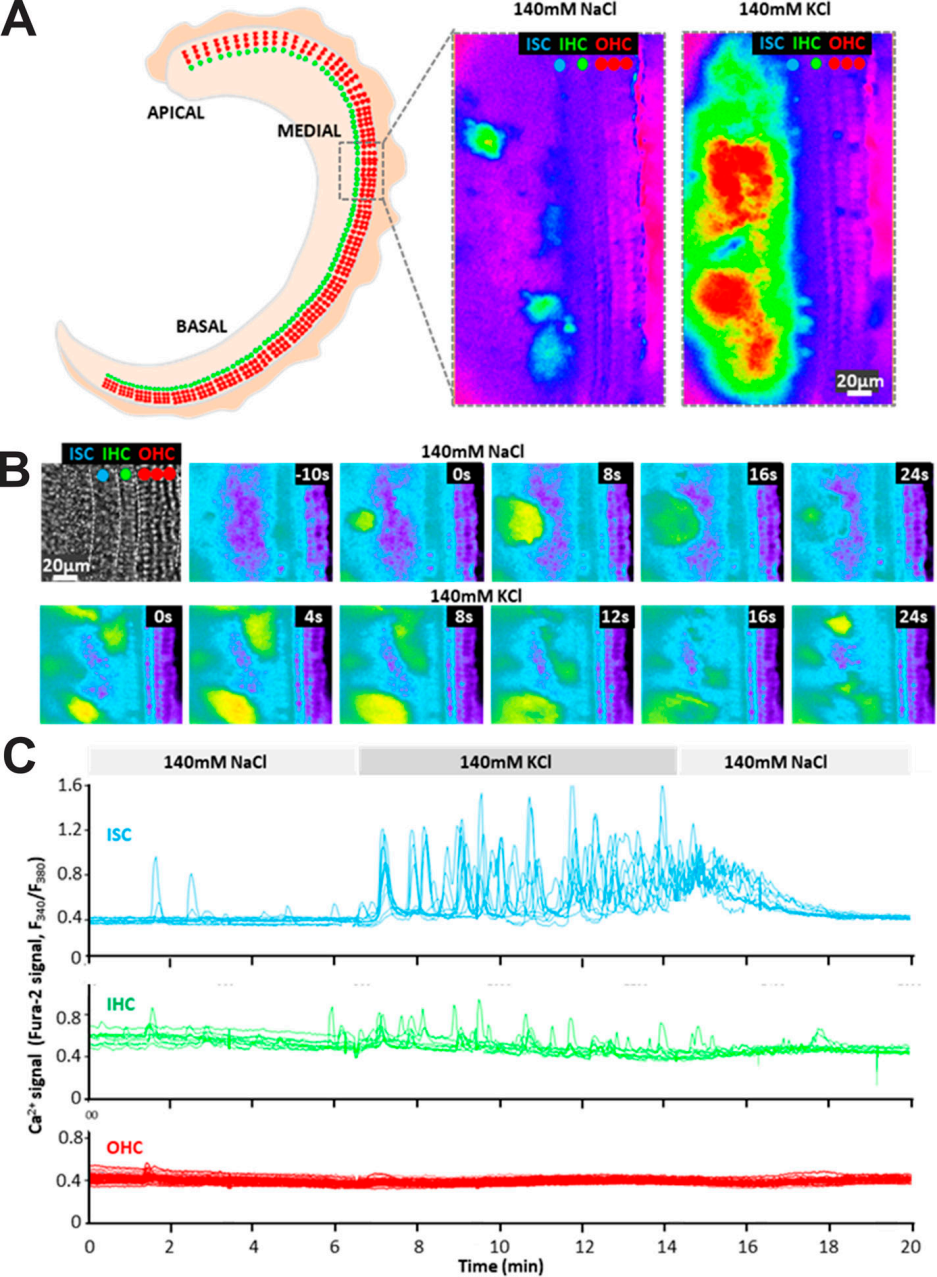
**Spontaneous Ca**^**2+**^ **wave activity in postnatal cochlear explants is increased by conditions that increase Cx hemichannel opening. (A)** Illustration of a cochlea explant extending from apical to basal regions. Recordings of Ca^2+^ activity were made in the apical/medial region of a mouse cochlear explant extracted at P7. Shown is a snapshot (image) of relative Ca^2+^ levels, evaluated the F_340_/F_380_ ratio of Fura-2 fluorescence, with the explant bathed in 140 mM NaCl containing 0 added extracellular Ca^2+^. Several regions of spontaneous Ca^2+^ activity were evident. When KCl was substituted for NaCl, which causes depolarization and further opening of Cx hemichannels, the Ca^2+^ activity was robustly enhanced, showing extensive areas of elevated Ca^2+^. **(B)** A sequence of images over time from the same cochlear explant shows the dynamics of Ca^2+^ wave activity. The top and bottom time sequences were recorded in 140 mM NaCl and 140 mM KCl, respectively; both solutions contained 0 added extracellular Ca^2+^. The top panel shows a transmitted light image of the cochlea explant and the cells in the designated regions, inner supporting cells (ISC, blue), inner hair cells (IHC, green) and outer hair cells (OHCs, red). **(C)** Recordings of Ca^2+^ activity from regions of interest positioned in the designated areas (ISC, IHC, and OHC) show the dramatic increase in the frequency and amplitude of the Ca^2+^ activity upon switching to 140 mM KCl.

**Figure S7.**
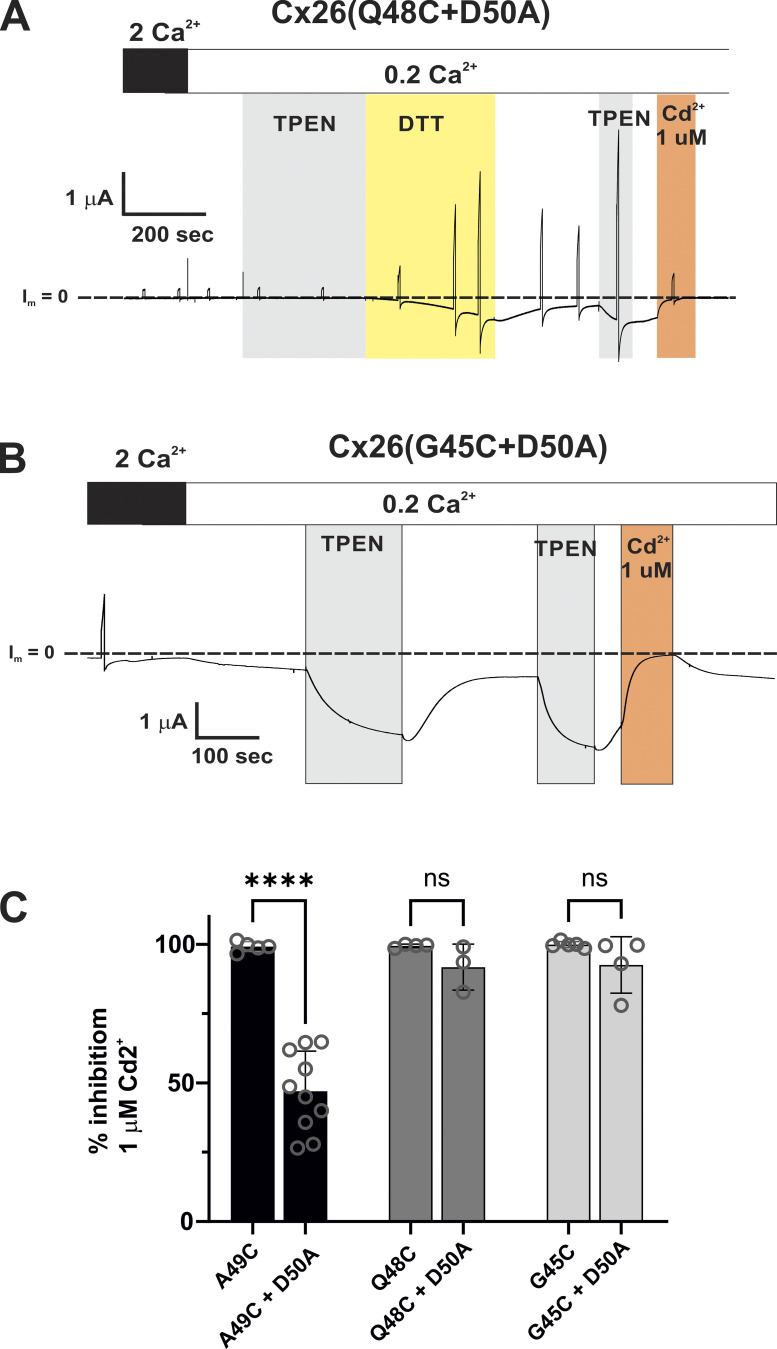
**Neutralization of D50 does not generally impact the effects of TPEN and Cd**^**2+**^ **with Cys substitutions at parahelical residues. (A and B)** Shown are examples of the effects of TPEN and 1 μM Cd^2+^ in A Cx26(Q48C+D50C) hemichannels and (B) Cx26(G45C+D50A). Cys substitutions at Q48 and G45 both show high-affinity metal binding evidenced by strong potentiation of currents by TPEN and robust inhibition by 1 μM Cd^2+^. These properties are retained when D50 is neutralized to D50A. **(C)** Summary of data comparing inhibition of hemichannels by 1 μM Cd^2+^ for A49C and Q48C substitutions, with and without neutralization of D50. Open circles represent results individual data points. Bars represent mean % inhibition ±SD. Significance among selected groups was tested using a one-way ANOVA and post-hoc Tukey’s adjusted for pairwise comparison. Neutralization of D50 only significantly weakened the effects of 1 μM Cd^2+^ with Cys substitution at A49. Asterisks denote statistical significance (****P value <0.0001; ns denotes no significance). P values for Q48C versus Q48C+D50A (0.6693); G46C versus G46C+D50A (0.6131). A49C (*n* = 6), A49C = D50A (n-10), Q48C (*n* = 5), Q48C+D50A (*n* = 4); G45C (*n* = 5), G45C+D50A (*n* = 4).

**Figure S8.**
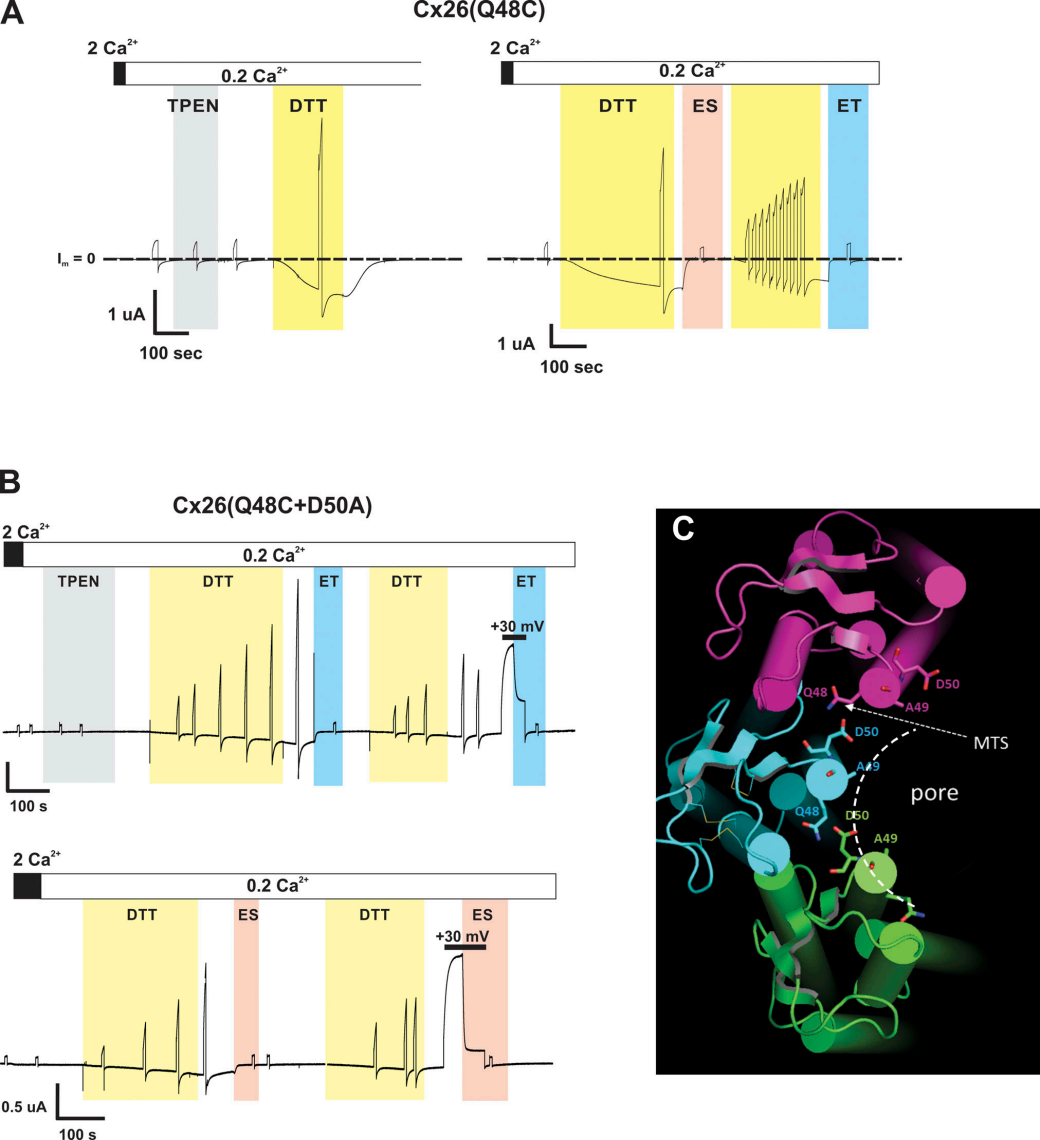
**Effects on Cx26 hemichannels upon Cys substitution at Q48 is unaffected by neutralization of D50.** Shown are recordings of currents from oocytes expressing Cx26(Q48C) and Cx26(Q49C+D50A). Cells were voltage clamped at −40 mV bathed in low (0.2 mM) Ca^2+^. Small test pulses to +30 were periodically applied to better illustrate activation. No hemichannel currents were activated in low Ca^2+^. The baseline current (designated as I_m_ = 0) is indicated by the dotted line. **(A)** For Cx26(Q48C) hemichannels, application of 10 μM TPEN (shaded gray area) was ineffective at inducing currents, whereas 200 μM DTT (shaded yellow area) led to activation (left trace). Right trace shows results of MTS reagents applied in succession (DTT reverses the covalent MTS modification by reducing the formed disulfide). Both MTSES and MTSET led to a robust decline in current. **(B)** For Cx26(Q48C+D50A) application of 10 μM TPEN remained ineffective at inducing current (top trace). Following activation and washout of DTT, the application of MTSET (top trace) and MTSES (bottom) trace continued to produce a robust decrease in current. Sequential MTS applications are shown, one at −40 mV and the other at +30 mV (indicated the bar) to illustrate the robustness of the effect. **(C)** Representation of three of six connexin subunits in a Cx26 hemichannel around a central aqueous pore using the 2ZW3 atomic structure of the Cx26 GJ channels (Maeda et al., 2009). Each subunit is represented by a different color. Cylinders represent the transmembrane domains. For each of the subunits Q48, A49, and D50 residues are shown. The side chains of A49 and Q48 are in close proximity to D50; the latter is intersubunit. Although not depicted as openly exposed to the pore in the structure, MTS reactivity indicates that Q48C is accessible with a likely path from the aqueous pore. The structures is displayed using PyMOL software (https://www.pymol.org).

Fig. S1 demonstrates that the neutralization of D50 to a Glu residue (D50N) results in the same effects as neutralization to an Ala residue (D50A). Fig. S2 shows that hemichannels carrying A49E and A49K substitutions are insensitive to DTT/TPEN and 1 uM Cd^2+^. Fig. S3 summarizes data on levels of Cx26 and Cx30 hemichannel currents in *Xenopus* oocytes at three different extracellular Ca^2+^ concentrations. Fig. S4 summarizes data showing that Cx30 hemichannels with mScarlet attached to its C-terminus behave the same as WT Cx30 hemichannels with respect to functional efficiency and effects of neutralization of the charge pair at positions 49/50. Fig. S5 shows examples of effects of MTSET and MTSES applications in Cx26 and Cx30 hemichannels with Cys-substitutions at positions 49 alone and in combination with position 50 neutralized to an Ala. Fig. S6 illustrates that spontaneous Ca^2+^ wave activity in the inner supporting cells in mouse cochlear explants increases robustly when exposed to low extracellular Ca^2+^ and high extracellular K^+^, conditions that robustly promote Cx hemichannel opening. Fig. S7 illustrates that unlike A49C hemichannels, Q48C and G45C hemichannels maintain a high sensitivity to Cd^2+^ following neutralization of D50 suggesting that high-affinity metal coordination in A49C hemichannels involves coordination between the introduced Cys side chain at position 49 with the carboxylate of the Asp side chain at position 50. Fig. S8 shows examples of MTS effects on Q48C hemichannels with and without neutralization of D50. Also shown is the positioning of Q48, A49, and D50 residues relative to the pore based on the PDB ID 2ZW3 crystal structure of the Cx26 GJ channel.

## Results

**Figure 1.**
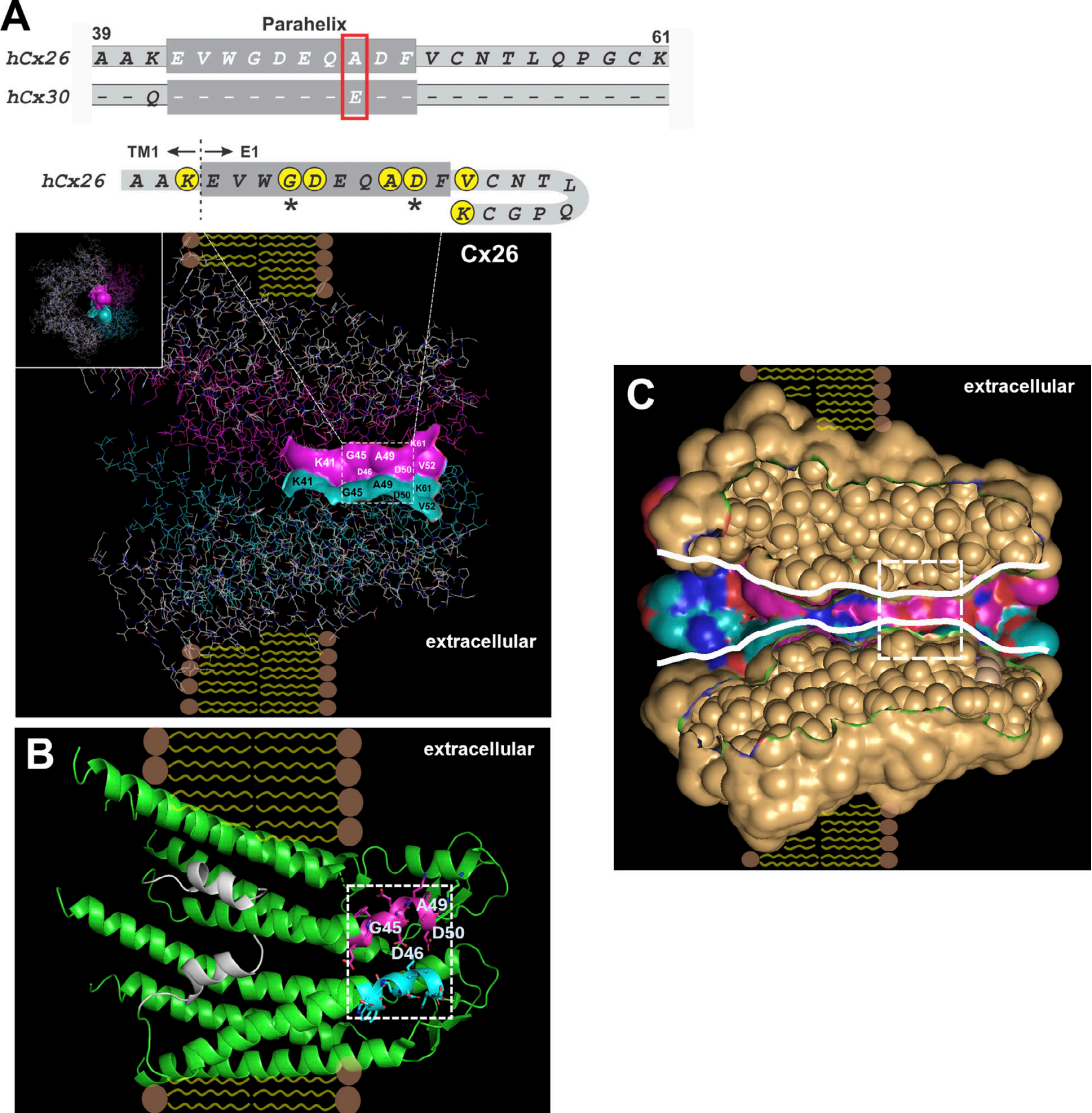
**The proposed pore-lining parahelical segment of the E1 domain differs at a single position between Cx26 and Cx30. (A)** Shown are sequence alignments of Cx26 and Cx30 from residues A39-K61 which extends from the TM1/E1 border and into the proximal portion of E1. The wider shaded region (darker gray) depicts the parahelix proposed by computational studies of Cx26 (Kwon et al., 2012). The sequences of Cx26 and Cx30 within the parahelix are identical (indicated by dashed lines) except for an Ala/Glu difference at position 49 (red boxed residues). A structural depiction of this region is illustrated in one hemichannel of a Cx26 GJ and is shown along with the sequence for Cx26 displayed with the E1 domain looping back as indicated by the structure. Two of six subunits are absent to enable viewing of the aqueous pore en face. Of the four displayed subunits, two adjacent subunits are colored (magenta and cyan) and show the predicted solvent-accessible residues in E1 using surface-rendering. The predicted pore-lining residues within this segment are also shown in the accompanying Cx26 sequence and are identified by filled circles (yellow); two circled residues indicated by asterisks were verified in previous studies as pore-lining in undocked hemichannels. **(B)** The same two subunits are displayed in a cartoon view highlighting the parahelical region (magenta and cyan). **(C)** A cut-away view of a surface representation of a Cx26 hemichannel illustrates the approximate contours of the pore extending from extracellular to intracellular ends. The parahelix aligns within a narrowed region of the pore (white-boxed region). The approximate membrane boundaries are illustrated as depictions of lipid bilayers. Structures are displayed using PyMOL software (https://www.pymol.org) and the PDB ID 2ZW3 coordinates of the Cx26 GJ channel (Maeda et al., 2009).

The primary sequences of the presumed parahelical regions and flanking residues of Cx26 and Cx30 near the TM1/E1 border are shown in Fig. 1 along with a structural view of the pore based on the PDB ID 2ZW3 crystal structure of the Cx26 GJ channel (Maeda et al., 2009). The pore-lining residues in two adjacent subunits are shown in a color-rendering of the solvent-accessible surfaces along with a cartoon view of the same two subunits. The parahelical segment, based on computational studies of Cx26, is proposed to extend from E42 through F51 (Kwon et al., 2012) and contains four residues, G45, D46, A49, and D50, predicted to be pore-lining in the original crystal structure. Previous studies using the substituted cysteine accessibility method (SCAM) identified G45 and D50 as pore-lining residues in functionally active Cx26 hemichannels (Sánchez et al., 2010; Sanchez et al., 2013). Both these residues are positions at which KID syndrome mutations have been identified (reviewed in Lee and White [2009]). A depiction of the overall approximate shape of the pore is provided in a cut-away view of a surface representation of the Cx26 structure and shows wide vestibules at cytoplasmic and extracellular ends and a narrower region that aligns with the position of the parahelix (white boxed region). Computational studies indicate a pore diameter of ∼15 Å in the parahelical region in the open conformation (Bargiello et al., 2018).

### Cys substitution at A49, disulfide bond formation, and high-affinity metal coordination

We made a Cys substitution at A49 in Cx26, a putative pore-lining residue within the parahelix that has not been functionally tested. As in previous SCAM studies, Cys-substituted variants were constructed and expressed in *Xenopus* oocytes. Hemichannel currents were examined in solutions containing 2.0 or 0.2 mM extracellular Ca^2+^; application of 0.2 mM Ca^2+^ consistently induced robust activation of Cx26 hemichannels. However, Cx26(A49C) hemichannels showed no evidence of functional currents. Given that introduced Cys residues could participate in the formation of disulfide bonds or the coordination of thiophilic metals that can render hemichannels non-functional, DTT was applied (100–200 µM), which acts both as a reducing agent and as a chelator of metal ions. Indeed, DTT was able to induce function in A49C hemichannels.

**Figure 2.**
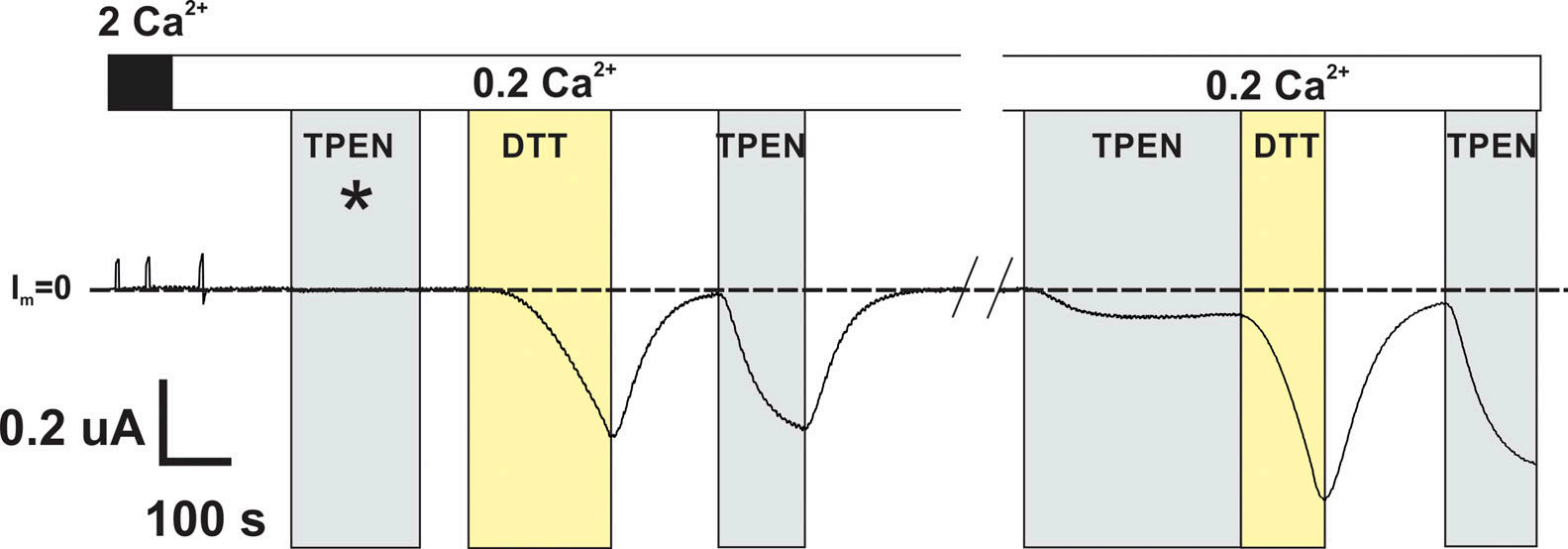
**Cysteine substitution at A49 in Cx26 impairs hemichannel function due to disulfide bond formation as well as metal ion coordination**. Shown is a recording of hemichannel current from a Cx26(A49C)-expressing *Xenopus* oocyte exposed to a sequence of TPEN- or DTT-containing solutions. The cell was voltage-clamped at −40 mV. Shaded areas indicate periods of application of either 10 µM TPEN (gray) or 200 µM DTT (yellow) in the continued presence of low (0.2 mM) Ca^2+^. No hemichannel current was evident in high (2 mM) Ca^2+^ or upon exposure to 0.2 mM Ca^2+^; baseline current at −40 mV (designated as I_m_ = 0) is indicated by the dashed line. Application of TPEN was initially ineffective at inducing current (asterisk), but following application of DTT, which reliably and effectively induced large inward currents, TPEN alone became effective. The effectiveness of TPEN diminished over time following DTT exposure and washout suggesting that Cys at position 49 can participate in disulfide bond formation and that following reduction and hemichannel opening can transiently participate in metal coordination. The break in the recording time shown represents ∼5 min.

To further explore the DTT-sensitive Cx26(A49C) currents, the effects of DTT were compared with TPEN, a strong chelator of transition metals with no reducing capacity. Application of TPEN (10 µM) for several minutes to naïve cells expressing Cx26(A49C) typically was ineffective at inducing currents. In contrast, the application of DTT (100–200 µM) reliably induced substantial currents, but they declined back to baseline upon washout of DTT. However, if TPEN was applied following application and washout of DTT, then TPEN alone became effective at inducing currents. An example of a sequence of TPEN and DTT applications to Cx26(A49C) hemichannels is shown in Fig. 2. Starting with TPEN, no current activation was evident during an ∼3-min application in 0.2 mM Ca^2+^. Following the washout of TPEN, a large current was elicited with the application of DTT. After washout of DTT, the current returned to baseline, but subsequent application of TPEN alone now rapidly induced a large current suggestive that a pool of hemichannels remained available for activation by metal chelation following activation by DTT. Although variable in extent and degree, longer intervals between application and washout of DTT and subsequent application of TPEN tended to reduce the ability of TPEN alone to initiate opening. The reduced effectiveness of TPEN is shown for a 5-min interval following the preceding TPEN application. The current elicited by TPEN after this ∼5 min interval was considerably smaller than the previous application. Subsequent DTT and then TPEN applications again elicited large currents, indicative that Cx26(A49C) hemichannels remained expressed in the membrane. These data indicate that Cx26(A49C) hemichannel function can be inhibited due to the formation of disulfide bonds as well as the coordination of thiophilic metals. The effects of TPEN and DTT when applied together did not differ from application of DTT alone (not shown), indicating that both agents acted on mitigating the effects of the A49C substitution.

**Figure 3.**
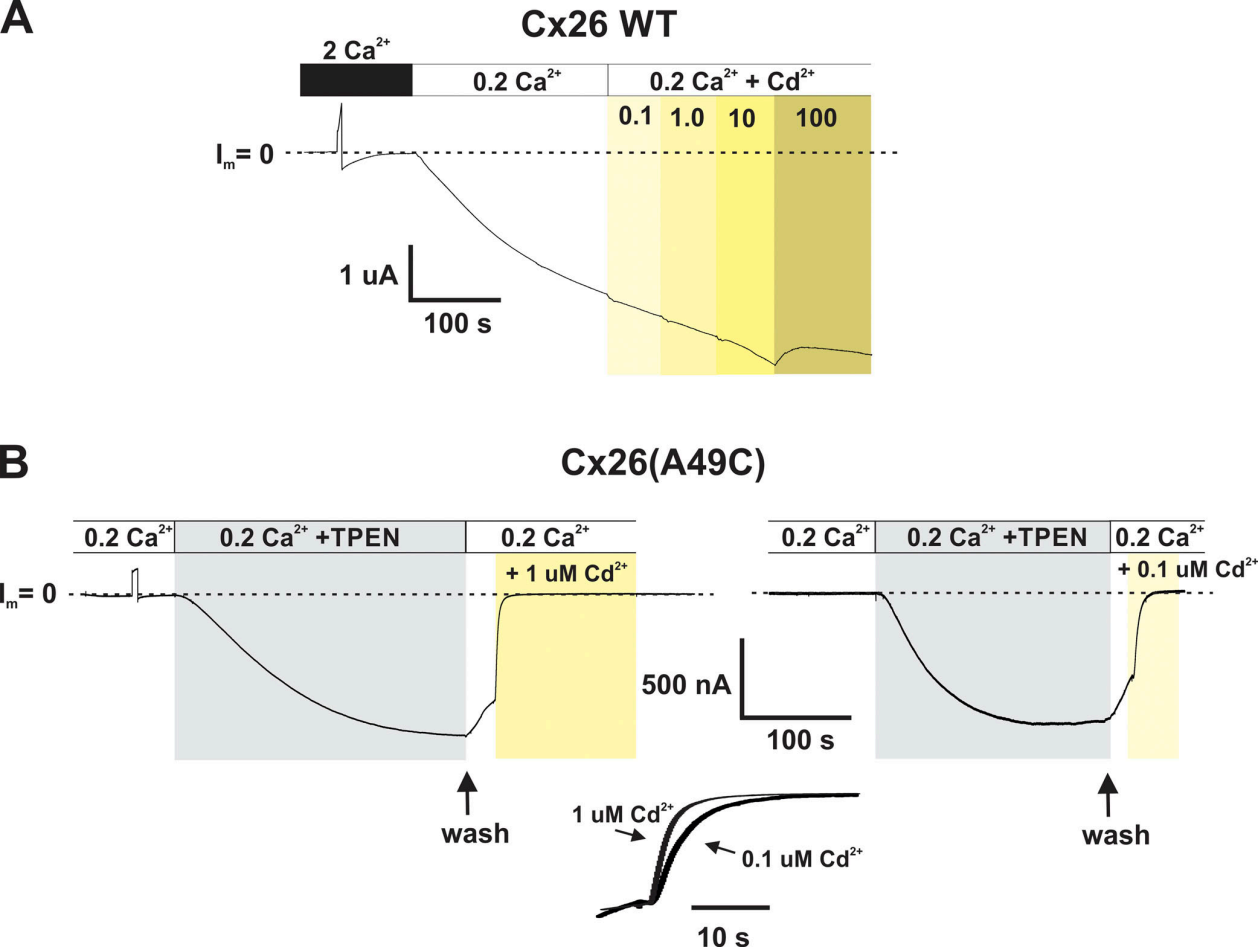
**Cysteine substitution at A49 in Cx26 hemichannels creates a high-affinity metal coordination site. (A)** Recording of hemichannel currents from a Cx26-expressing *Xenopus* oocyte. The cell was voltage-clamped at −40 mV. Exposure to low (0.2 mM) Ca^2+^ produced a large inward current; the baseline current (designated as I_m_ = 0) is indicated by the dashed line. Sequential additions of Cd^2+^ concentrations ranging from 0.1 to 10 µM (indicated by shaded areas) in the continued presence of 0.2 mM Ca^2+^ had no effect on the developing current. Some evidence of modest inhibition was observed with application of 100 μM Cd^2+^. **(B)** Recordings of hemichannel currents from a Cx26(A49C)-expressing oocyte. Following activation by TPEN in low (0.2 Ca^2+^), a decline in current was evident upon washout of TPEN (arrows), but rapidly accelerated when switching to wash solutions containing Cd^2+^. Shown are effects of 1 and 0.1 μM concentrations of Cd^2+^, both of which were ineffective in WT Cx26 hemichannels. Superimposition of the time courses of the Cd^2+^-induced reductions for 1.0 and 0.1 µM concentrations are shown in an expanded time scale and shows a concentration dependence of the time course of inhibition. DTT was applied and washed out prior to the illustrated TPEN applications. Flow rates of wash solutions with and without Cd^2+^ were the same.

The return of Cx26(A49C) hemichannel currents to baseline following washout of DTT or TPEN suggests that nominal levels of metals present in the solutions and/or the cells are sufficient to inhibit hemichannel function, consistent with the creation of a high-affinity, metal-coordination site upon introducing a Cys residue at position 49. As confirmation, the effects of bath application of the thiophilic metal Cd^2+^ were examined following induction with TPEN. WT Cx26 hemichannels are largely insensitive to Cd^2+^ concentrations as high as 100 µM (Fig. 3 A). However, a 1,000-fold lower concentration of Cd^2+^ produced rapid inhibition of Cx26(A49C) hemichannel currents. Examples are shown for 1.0 and 0.1 μM Cd^2+^ concentrations (Fig. 3 B) applied during the washout phase of TPEN to distinguish effects caused by the applied Cd^2+^ from those caused by the nominal levels of metals present in the wash and/or cells; the cells had been previously exposed to DTT prior to TPEN application. For both concentrations of Cd^2+^, the rate of decline toward baseline increased substantially upon the addition of Cd^2+^ indicating that submicromolar concentrations of Cd^2+^ can bind and impede current flow in Cx26(A49C) hemichannels.

### Modification of A49C by MTS reagents results in robust inhibition of Cx26 hemichannels

**Figure 4.**
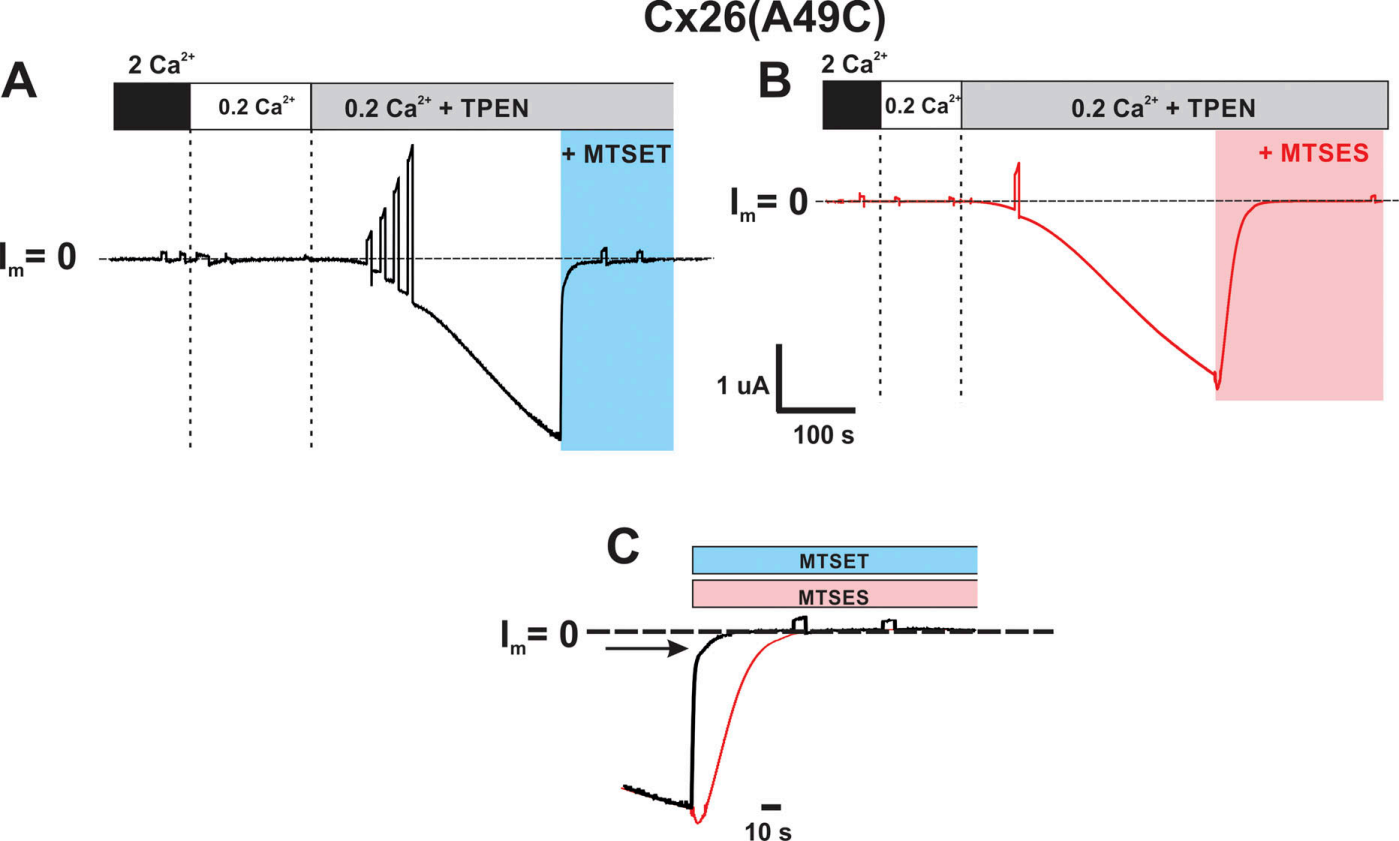
**A49C is modified by charged MTS reagents and results in robust inhibition of Cx26 hemichannels.** Shown are recordings of hemichannel currents from *Xenopus* oocytes expressing Cx26(A49C) that were previously exposed to DTT. The cells were voltage clamped at −40 mV. Following induction of current with TPEN in low (0.2 mM Ca^2+^) MTS, reagents were applied in the continued presence of TPEN; baseline currents at −40 mV (designated as I_m_ = 0) are indicated by the dashed lines. **(A)** Application of MTSET (0.2 mM) led to a rapid and robust decline in current to baseline (blue shaded area). **(B)** Application of MTSES (2 mM) also led to a rapid and robust decline in current to near baseline (red shaded area) except for a small increase in current that preceded the decline. For both reagents, the current remained at baseline following washout of MTSET in the continued presence of TPEN (not shown) consistent with covalent modification by the MTS reagents. **(C)** The time courses following the application of MTSET or MSTES are illustrated by the superimposition of recordings on an expanded time scale. MTSES effects were slower than MTSET effects as expected, in large part, from the lower rate constant for thiol reaction (Karlin and Akabas, 1998). A biphasic response to MTSES is evident as a rapid increase in current that precedes the decline to baseline. Although not quantified, the response to MTSET also appeared biphasic with a rapid decrease in current preceding a slower decline to baseline (the slow phase is indicated by the arrow).

Next, we assessed whether A49C is exposed to the pore by testing for accessibility to modification by thiol-modifying reagents in Cx26 hemichannels. The positively and negatively charged methanethiosulfonate (MTS) reagents MTSET and MTSES were used because of their membrane impermeability and fast reaction times with aqueous-exposed Cys residues in their thiolate form Karlin and Akabas (1998). As demonstrated previously, these MTS reagents readily enter Cx hemichannel pores and produce rapid effects on currents when modifying pore-lining residues (Kronengold et al., 2003; Sánchez et al., 2010; Sanchez et al., 2013; Verselis, 2009). Typically, modifications by MTSES and MTSET produce opposite effects, rapid increases and decreases in current, respectively, reflecting opposite biases on net ionic fluxes upon introduction of oppositely charged side chains into the permeation pathway. For Cx26(A49C) hemichannels, however, application of either MTSET or MTSES following activation in low (0.2 mM) Ca^2+^ produced robust inhibition of currents, even in the continued presence of TPEN to prevent metal-ion coordination. These results are illustrated with characteristic examples (Fig. 4, A and B). Closer inspection of the MTS effects showed that prior to inhibition, a small, transient increase in current was observed with MTSES suggesting two phases of effects. Although less distinctive, MTSET also appeared to produce two phases of effects, but of the same sign with a rapid decrease followed by a slower decrease to baseline. These biphasic effects are illustrated in superimposed recordings shown at a higher time resolution (Fig. 4 C).

### Interactions between positions 49 and 50 can robustly affect Cx26 hemichannel function

**Figure 5.**
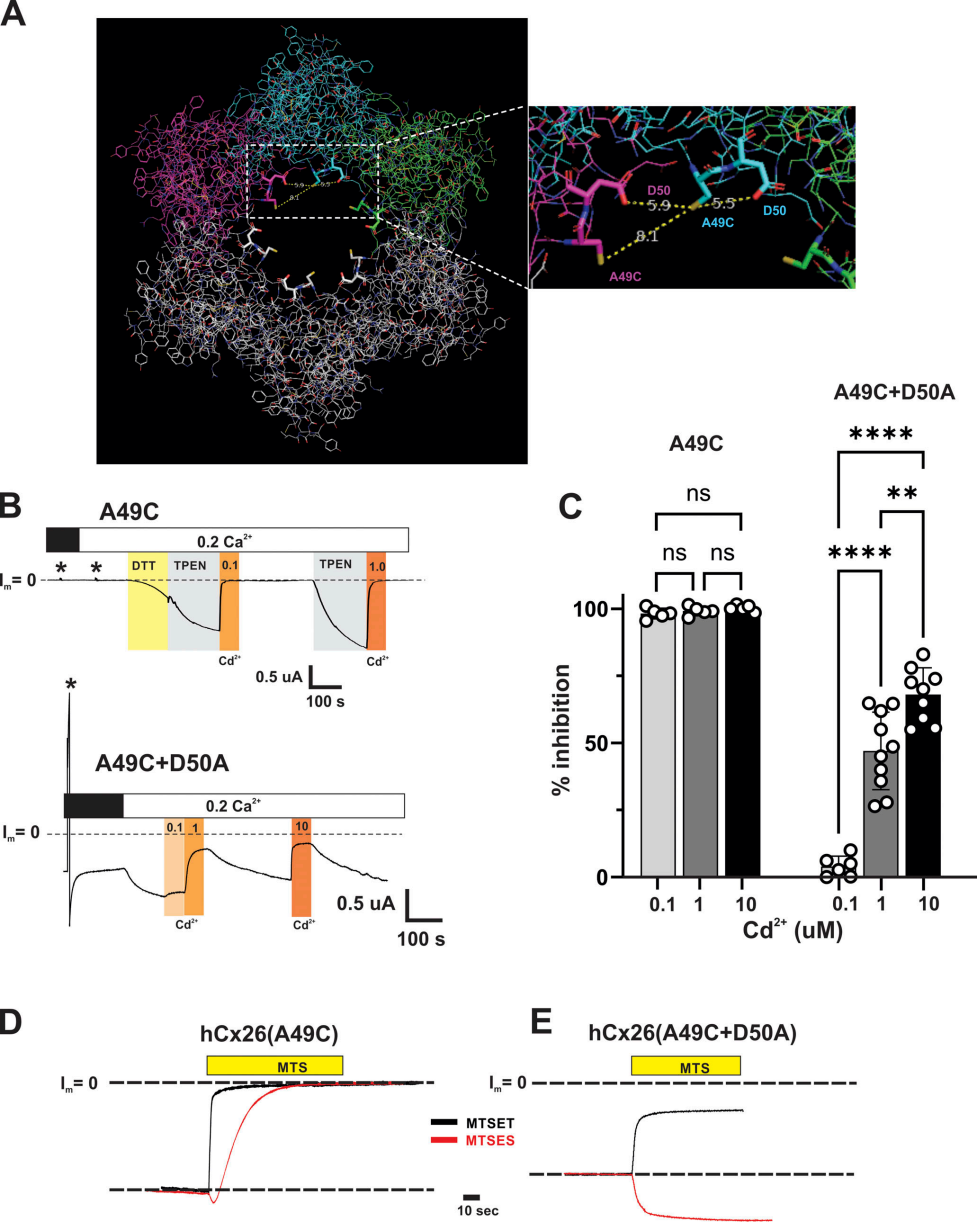
**Cx26 hemichannels with the A49C substitution show markedly reduced sensitivity to Cd**^**2+**^ **and altered effects of MTS reagents upon neutralization of the adjacent D50 residue. (A)** View of a Cx26 hemichannel down the aqueous pore from the extracellular side. Three adjacent subunits colored in magenta, cyan, and green highlight potential intra- and inter-subunit interactions involving positions 49 and 50 with A49 modified to a Cys (see the expanded view from the boxed region). The structures are displayed using PyMOL software (https://www.pymol.org) from the PDB ID 2ZW3 coordinates of the Cx26 GJ channel (Maeda et al., 2009). Residues 1–41 are excluded to allow for better visualization of the E1 region of the pore. The position of the A49C side chain represents the rotamer with the predicted highest frequency of occurrence. **(B)** Examples showing the different sensitivities to exogenously applied Cd^2+^ for A49C+D50A hemichannels (bottom recording) compared to A49C hemichannels (top recording). Baseline currents levels (designated I_m_ = 0) are indicated by dashed lines. Asterisks denote currents in response to brief (5 s) voltage steps to +40 mV prior to DTT and TPEN applications. A49C shows no evidence of hemichannel current, but DTT followed by TPEN induced robust currents that were inhibited by a sub-micromolar concentration of Cd^2+^. Recovery of currents following washout of Cd^2+^ required reapplication of TPEN. In contrast, A49C+D50A hemichannels showed currents in 2.0 and 0.2 mM Ca^2+^ in the absence of applied TPEN or DTT. Application of Cd^2+^ showed inhibition, but with reduced sensitivity; 0.1 μM Cd^2+^ showed little effect. Also, currents recovered following washout of Cd^2+^ and did not require TPEN. **(C)** Summary of data for sensitivity to Cd^2+^ for A49C and A49C+D50A hemichannels. Open circles represent individual measurements of % inhibition (relative to the baseline current level) in different oocytes. The bars represent the mean ± SD. Significance among Cd^2+^ concentrations was tested using a one-way ANOVA and post-hoc Tuckey’s adjusted for pairwise comparisons. A49C hemichannels essentially exhibited total inhibition at all three concentrations tested with no significant differences. % inhibition differed for A49C+D50A hemichannels at all three concentrations. Asterisks denote statistical significance (**P value <0.01; ****P value <0.0001; ns denotes no significance—P values for pairwise comparisons of A49C were: 0.1 versus 1 μM (0.292); 0.1 versus 10 μM (0.056) and 1 versus 10 μM (0.525). The P value for A49C+D50A, 1 versus 10 μM was 0.0013. *n* ranged from 5 to 10 measurements at each concentration. **(D and E)** Examples of the changes in the effects of MTS modification of A49C upon neutralization of D50. The currents showing the effects of MTSET and MTSES are superimposed for (D) Cx26(A49C) and (E) Cx26(A49C+D50A) hemichannels. Currents were normalized to values preceding the MTS application. In Cx26(A49C+D50A) hemichannels, MTSET now produced a reduction in the current of ∼60%, and the effect of MTSES reversed in sign, showing only a sustained increase in current.

Considering the inhibition of currents upon modification of Cx26(A49C) hemichannels by charged MTS reagents and that Cd^2+^ coordination sites can consist of Cys residues and nearby Asp or Glu residues, examination of the Cx26 structure (Fig. 5 A) suggests that the adjacent D50 residue could play a role, potentially acting as a partner with A49C in coordinating Cd^2+^ and mediating electrostatic interactions following imposition of charge at A49C through MTS modification. Thus, a double mutant, Cx26(A49C+D50A), was constructed to remove the carboxylate at position 50 in an A49C background. In contrast to Cx26(A49C) hemichannels, double-mutant Cx26(A49C+D50A) hemichannels produced currents without the application of DTT or TPEN (Fig. 5 B). Inhibition by Cd^2+^ remained but was reduced in sensitivity (Fig. 5 C). In addition, the reductions in current with the application of Cd^2+^ to Cx26(A49C+D50A) hemichannels were readily reversible upon washout consistent with reduced or absent binding at endogenous/contaminant levels of metals. Taken together, these results are consistent with a disruption of the high-affinity metal coordination site in Cx26(A49C) hemichannels upon neutralization of D50, with the remaining effects of Cd^2+^ potentially reflecting retained lower-affinity, mono-dentate binding at A49C.

Notably, the D50A substitution dramatically changed the effects of MTS modification of A49C (Fig. 5, D and E). Although A49C remained accessible to MTS reagents in Cx26(A49C+D50A) hemichannels, modification by MTSET did not reduce the current to near baseline, but rather produced a decrease in current by about 60%. More remarkably, the net effect of MTSES reversed in sign, only producing a rapid and sustained increase in current (see also Fig. 8 for a summary of the MTS data). The same results were obtained with a D50N substitution on an A49C background (Fig. S1); both D50N and D50A are KID mutations, with D50N being the most common of all KID mutations (Mazereeuw-Hautier et al., 2007). These data suggest that the removal of an electrostatic interaction between positions 49 and 50 results in a loss of the secondary (slower-phase) effects that ultimately produce robust inhibition of Cx26(A49C) hemichannels upon modification with MTSET or MTSES. The loss of this secondary effect results in a full unmasking of the rapid effects on open channel conductance.

**Figure 6.**
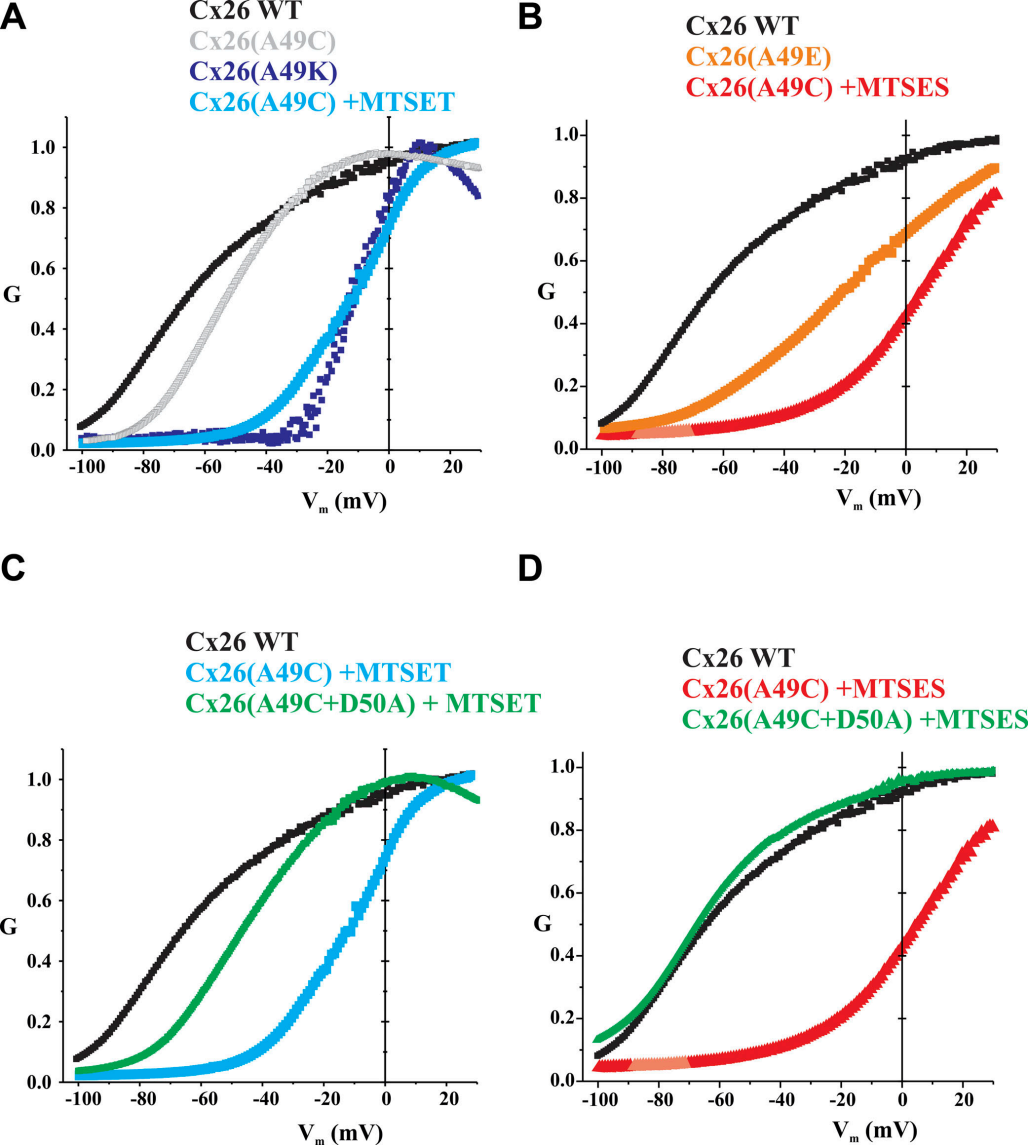
**Charge substitutions at A49 and MTS modifications of A49C produce robust shifts in the G-V relationships for Cx26 hemichannels positive along the voltage axis.** Shown are normalized average conductance–voltage (G-V) relationships for WT and modified Cx26 hemichannels in 0.2 Ca^2+^. Data were obtained by applying slow (600 s) voltage ramps from +40 to −100 mV from a holding potential of −20 mV. For each *Xenopus* oocyte, conductance was normalized to the maximum value. **(A)** Superimposed plots compare WT Cx26 hemichannels with those in which position 49 was changed to a positive charge either by an A49K substitution or an MTSET modification of A49C. Also included is A49C in TPEN without MTS modification. **(B)** Superimposed plots compare WT Cx26 hemichannels with those in which position 49 was changed to a negative charge either through an A49E substitution or an MTSES modification of A49C. **(C and D)** Superimposed plots show that the double mutant (A49C+D50A) lacks the robust positive shift along the voltage axis after modification with MTSET (C) or MTSES (D) compared with the single A49C mutant. The G-V plots represent means with error bars removed for easier visualization. *n* = 11 for WT Cx26 channels, 6 for Cx26(A49K) and Cx26(A49E), 5 for Cx26(A49C), and 4 for Cx26(A49C+D50A) modified with MTSET or MTSES.

Although application of either MTS reagent produced robust inhibition of Cx26(A49C) hemichannels, which we assessed at a holding potential of −40 mV, some modest degree of activation was evident upon depolarization to +40 mV suggesting that a positive shift in activation may be a contributing factor to inhibitory effects of MTS reagents we observed. Thus, we examined the G-V relationships of MTS-modified Cx26(A49C) hemichannels as well as Cx26 hemichannels containing A49E or A49K charge substitutions. Both the A49K and A49E substitutions produced functional hemichannels that were insensitive to DTT and TPEN (Fig. S2). Normalized G-V relationships in low (0.2 mM) Ca^2+^ indeed showed robust shifts in activation in the depolarizing direction (Fig. 6, A and B). Superimposed on these plots are normalized G-V relationships of both MTSET- and MTSES-modified Cx26(A49C) hemichannels, which also showed robust positive shifts in activation. MTSET modification of A49C produced a similar shift as an A49K substitution, but MTSES modification produced a larger shift than an A49E substitution. Although not rigorously quantified, the G-V relationships with positive charge at A49 appeared steeper, but the voltage at which conductance achieved half maximal was shifted more with negative charge than with positive charge at position 49. These shifts, in essence, reduce the tendency of Cx26 hemichannels to be open at resting membrane potentials, thereby contributing to the observed effects of MTS modification. Of note, the G-V relationship of A49C in the presence of TPEN, in the absence of MTS modification, showed a modest positive shift in activation.

### Cx30 hemichannel function

**Figure 7.**
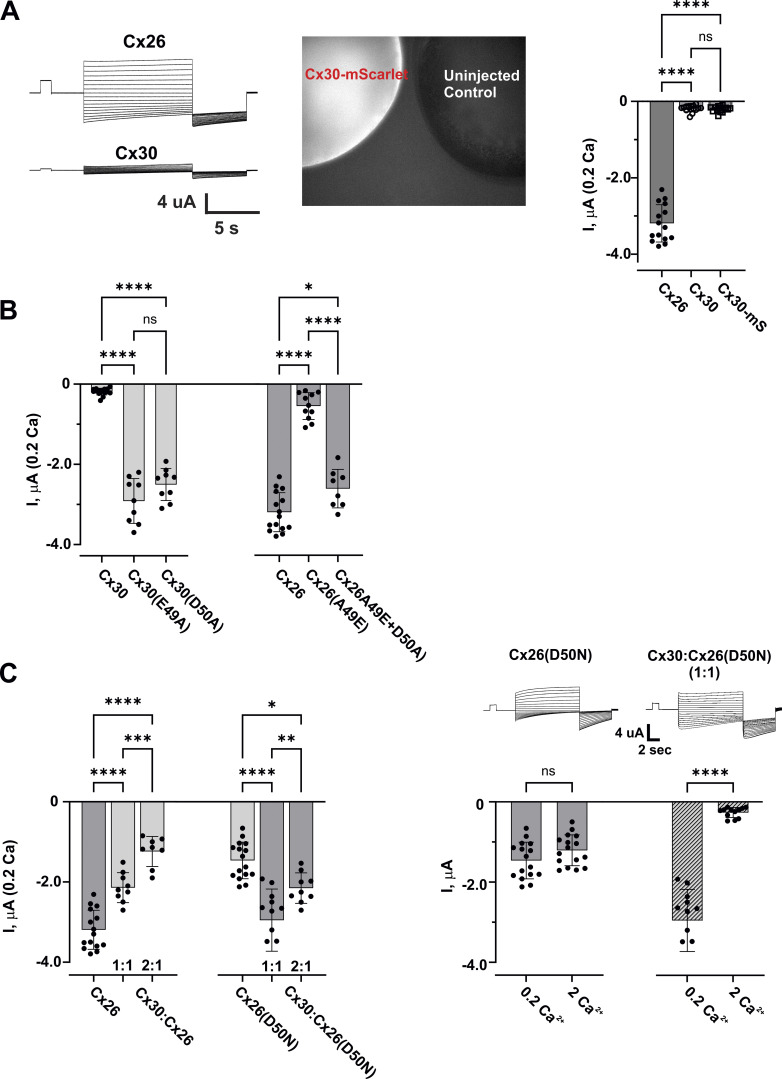
**Cx30 hemichannels exhibit reduced functionality. (A)** Bar graphs showing mean values of hemichannel currents measured at a holding potential of −40 mV in solutions containing 0.2 mM Ca^2+^ for WT Cx26, WT Cx30, and Cx30 with mScarlet fused to the C-terminus (Cx30-mS). Filled circles represent individual measurements in different oocytes. WT Cx26 hemichannels exhibited robust currents, whereas WT Cx30 and Cx30-mScarlet (Cx30-mS) hemichannels exhibited small currents marginally detectable above baseline. Examples of currents for WT Cx26 and WT Cx30 hemichannels are shown in response to a voltage step protocol consisting of a brief prepulse to +10 mV from a holding potential of −20 mV followed by steps ranging from +50 to −110 mV and then −110 mV before returning to −20 mV. Representative fluorescent image of an oocyte expressing Cx30-mScarlet adjacent to an uninjected control showing robust Cx30 expression despite exhibiting small currents (not shown). **(B)** Comparison of mean values of hemichannel currents for WT Cx30, Cx30(E49A), and Cx30(D50A) compared with WT Cx26, Cx26(A49E), and Cx26(A49E+D50A). For either Cx, currents were notably smaller with a negative charge pair at positions 49 and 50, as occurs naturally in Cx30. **(C)** Bar graphs showing mean values of hemichannel currents for oocytes co-injected with Cx26 and Cx30. Oocytes were injected with a 1:1 ratio of cRNAs or a 2:1 ratio favoring Cx30. The increasing presence of Cx30 dampened currents when co-expressed with WT Cx26, but not with Cx26(D50N), suggesting that neutralization of D50 in Cx26 subunits robustly improves Cx26:Cx30 heteromeric hemichannel functionality. Bar graphs on the right show currents for Cx26(D50N) alone compared with those injected 1:1 with Cx30:Cx26(D50N) in recording solutions containing 2.0 and 0.2 mM Ca^2+^. Currents in oocytes injected with 1:1 Cx30:Cx26(D50N) show a robust sensitivity to Ca^2+^ not present in cells injected with Cx26(D50N) alone consistent with heteromeric hemichannel formation. All oocytes were injected with the same concentrations of cRNA. Measurements were obtained between 24 and 48 h, post injection, at −40 mV. All bars represent mean ± SD. Significance among groups was tested using a one-way ANOVA and post-hoc Tukey’s adjusted for pairwise comparison. For comparison of Cx26(D50N) in 0.2 and 2 Ca^2+^, as well as 1:1 co-injected Cx30:Cx26(D50N), measurements for both Ca^2+^ concentrations were obtained in the same oocyte, and unpaired *t* tests were used to assess significance. Asterisks denote statistical significance (*P value <0.1; **P value <0.01; ***P value <0.001; ****P value <0.0001; ns denotes no significance—P value for Cx30 versus Cx30-mScarlet was 0.9882, for Cx30(A49E) versus Cx30(D50A) was 0.0503, for Cx26 versus Cx26(A49E+D50A) was 0.0133. For comparison of Cx26(D50N) in 0.2 and 2 Ca^2+^, unpaired *t* test shows no significance (P value 0.0979) whereas a paired *t* test (not shown) showed significance (P value <0.0001) indicative of a small but significant Ca^2+^ effect. Paired and unpaired *t* tests for 1:1 co-injected Cx30:Cx26(D50N) both showed significance (P values <0.0001) indicative of the strong sensitivity to Ca^2+^. WT Cx26 (*n* = 15); WT Cx30 (*n* = 16); Cx39-mScarlet (*n* = 16); Cx30(E49A) (*n* = 9); Cx30(D50A) (*n* = 9); Cx26(A49E) (*n* = 11); Cx26(A49E+D50A) (*n* = 8); Cx30:Cx26 1:1 (*n* = 9). Cx30:Cx26 2:1 (*n* = 8); Cx26(D50N) (*n* = 16); Cx30:Cx26(D50N) 1:1 (*n* = 12). Cx30:Cx26 2:1 (*n* = 9).

Compared with Cx26, oocytes injected with the same concentration of Cx30 mRNA typically exhibited low levels of current over a wide range of voltages (−110 to +40 mV) and extracellular Ca^2+^ concentrations. In 2 mM Ca^2+^, Cx30 did not reliably produce detectable currents above baseline and remained low when placed in low (0.2 mM) extracellular Ca^2+^, a condition that reliably elicited robust Cx26 hemichannel currents (Fig. 7 A). We note that substantial Cx30 hemichannel currents could be elicited when cells were placed in a nominal Ca^2+^ solution (no added Ca^2+^), but it was difficult to compare with Cx26 currents, which in nominal Ca^2+^ were often so large that they precluded reliable clamping of the cells at larger voltages using the cRNA concentrations and injected volumes used in this study to elicit Cx30 currents (Fig. S3). We also constructed Cx30 with mScarlet attached to its C-terminus to visualize expression. Oocytes injected with Cx30-mScarlet showed bright fluorescence indicative of high levels of Cx30 expression (Fig. 7 A), but just like WT Cx30 exhibited no or relatively low levels of associated currents in 2.0 or 0.2 mM Ca^2+^ (Fig. S4). Cx30 has a naturally occurring Glu at position 49 that could interact with the adjacent D50, much like Cx26 modified at position 49, potentially explaining the inherently smaller Cx30-mediated hemichannel currents. Thus, we examined Cx30 hemichannels in which E49 or D50 was individually neutralized to E49A and D50A, respectively. When exposed to 0.2 mM Ca^2+^ in the bath, both Cx30(E49A) and Cx30(D50A) expressed robust hemichannel currents compared with WT Cx30 (Fig. 7 B). The same results were observed with variants tagged with m-Scarlet (Fig. S4). Conversely, for WT Cx26, which normally exhibits large currents in 0.2 mM Ca^2+^, an A49E substitution reduced currents and neutralization of D50 in an A49E background (A49E+D50A) restored currents to robust levels (Fig. 7 B). In addition, hemichannel currents were examined when co-expressing WT Cx30 with WT Cx26 or with the Cx26(D50N), the most common KID syndrome mutant, in equal ratios of cRNA concentrations or biased (2:1) toward Cx30. Although substantial hemichannel currents were evident with both mixtures, co-expressing WT Cx30 with WT Cx26 led to smaller currents compared with WT Cx26 alone; a greater reduction in current occurred with increased relative levels of Cx30. For Cx26(D50N), co-expression with Cx30 did not reduce currents but rather produced larger currents than Cx26(D50N) alone (Fig. 7 C). That co-injection of Cx30 and Cx26(D50N) resulted in heteromers is evident from the strong sensitivity to extracellular Ca^2+^ as Cx26(D50N) hemichannels alone have been shown to be relatively insensitive to Ca^2+^ (Lopez et al., 2013; Sanchez et al., 2013) and Cx30 hemichannels alone, as we show here, do not show appreciable currents under these Ca^2+^ conditions (Fig. 7 C). Thus, Cx30/Cx26 heteromers with reduced numbers of subunits containing D50 produce larger currents consistent with reductions in opportunities for electrostatic interactions between E49 and D50 being favorable for hemichannel function.

**Figure 8.**
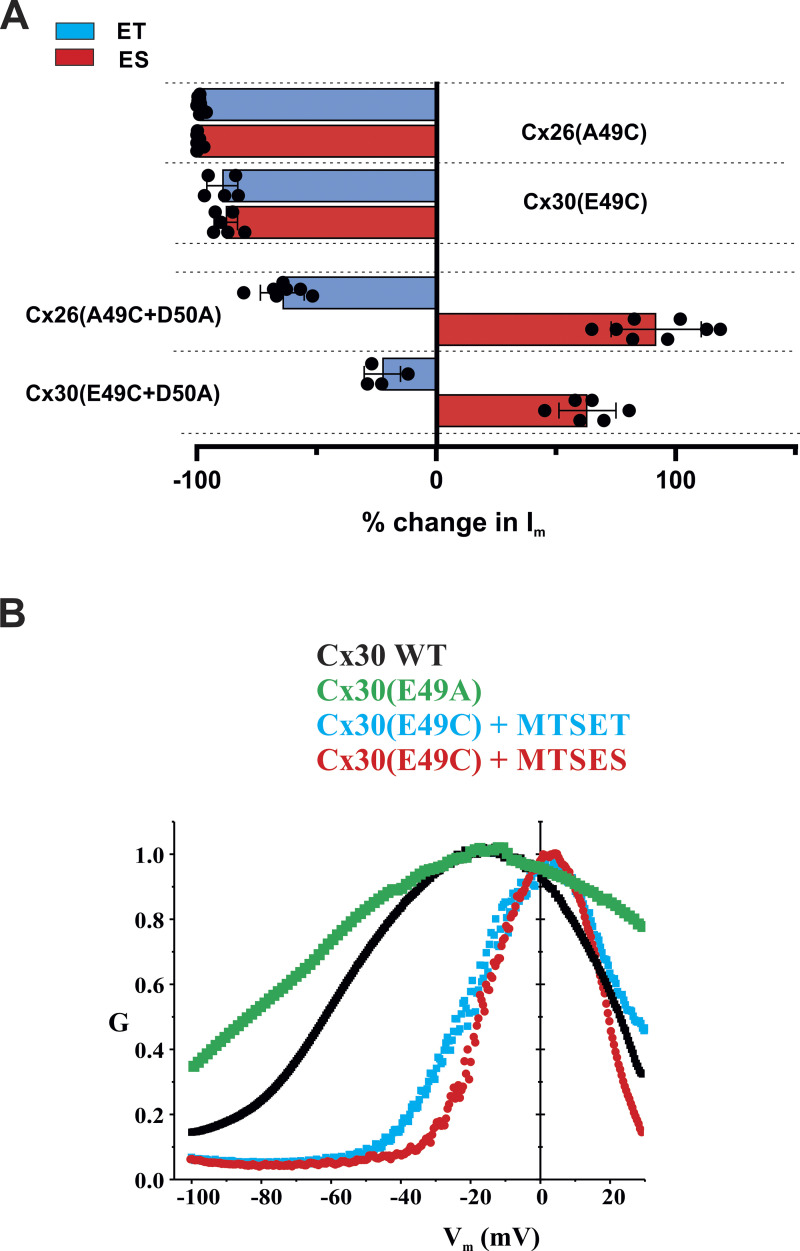
**E49 modifications in Cx30 show differences, but similar trends as A49 modifications in Cx26. (A)** Bar graphs showing % change in macroscopic currents for Cx26(A49C), Cx30(E49C), Cx26(A49C+D50A), and Cx30(E49C+D50A) hemichannels in response to bath application or MTSET (0.2 mM) or MTSES (2 mM). For Cx26(A49C) and Cx30(E49C), currents were induced with DTT+TPEN followed by TPEN alone. Filled circles represent individual measurements in different oocytes. MTS reagents were applied in the presence of TPEN at a holding potential of −40 mV. Application of MTSET and MTSES to Cx30(E49C) hemichannels led to robust inhibition, much like A49C in Cx26. Neutralization of D50 to D50A markedly changed the effects of modification by MTS reagents, most notably reversing in sign and showing only an increase in current with MTSES. Also of note, the magnitudes of the effects particularly for MTSET, were smaller in Cx30(E49C+D50A) than in Cx26(A49C+D50A) hemichannels. Each bar represents the mean ± SD obtained at −40 mV. Circles represent individual data points. Cx26(A49C)+ET (*n* = 9); Cx26(A49C)+ES (*n* = 6); Cx30(E49C)+ET (*n* = 5); Cx30(E49C)+ES (*n* = 6); Cx26(A49+D50A)+ET (*n* = 7); Cx26(A49+D50A)+ES (*n* = 8); Cx30(E49C+D50A)+ET (*n* = 4); Cx30(E49C+D50A)+ES (*n* = 6). **(B)** Shown are normalized average conductance–voltage (G-V) relationships for WT and modified Cx30 hemichannels in 0.2 Ca^2+^. Data were obtained by applying slow (600 s) voltage ramps from +40 to −100 mV from a holding potential of −20 mV. For each oocyte, conductance was normalized to the maximum value and superimposed on the same plot to compare WT Cx30 hemichannels with those in which E49 was neutralized to and Ala in which E49C was modified to a positive or negative charge with MTSET and MTSES, respectively. E49C hemichannels were activated by DTT+TPEN and then maintained in TPEN. The G-V plots represent means with error bars removed for easier visualization. WT Cx30 (*n* = 6); Cx30(E49A) (*n* = 7); Cx30(E49C) + MTSET (*n* = 5); Cx30(E49C) + MTSES (*n* = 5).

Given the potential impact of E49 on Cx30 hemichannel function, we wanted to check if position 49 in Cx30 is exposed to the pore, as it is in Cx26, and whether MTS reagents produce similar effects on hemichannel currents. Indeed, this was the case. No functional currents were evident for Cx30(E49C) hemichannels but could be activated with bath application of DTT and subsequently TPEN. Following activation, the application of MTSET or MTSES produced robust reductions in current with a transient increase evident with MTSES, but not MTSET. Moreover, the effect of MTSES reversed in sign upon neutralization of D50. One notable difference, however, was a smaller reduction in current upon modification with MTSET. In Cx30(E49C) hemichannels, the current did not decline as close to baseline with MTSET as in Cx26(A49C) hemichannels and Cx30(E49C+D50A) hemichannels, MTSET produced a relatively small (∼20%) reduction in current. Fig. 8 A summarizes the results of MTS modifications in both Cx26 and Cx30 hemichannels. Examples of recordings used to obtain data on MTS effects are shown for all single and double mutants in Fig. S5.

Although hemichannel currents for WT Cx30 were smaller in magnitude, the normalized G-V relationship was not substantially shifted positively along the voltage axis as might be expected due to the presence of an E49–D50 charge pair (Fig. 8 B). However, neutralization of E49 to A49 did cause a moderate hyperpolarizing shift and Cx30(E49C) hemichannels shifted substantially in a depolarizing direction upon modification with MTSET or MTSES. Thus, manipulations at position 49 in Cx30 hemichannels showed similar trends as those in Cx26 hemichannels, but there were differences between the two hemichannels suggesting that structural differences are sufficient to differentially impact the effects of residues at positions 49 and 50 on hemichannel gating and functionality. These normalized G-V relationships were examined in 0.2 mM Ca^2+^ to allow comparison upon neutralization of E49; much like for WT Cx26, Cx30(E49A) currents in nominal (0 added) Ca^2+^ were too large and often the cells were comprised and difficult to clamp. Also of note, Cx30 hemichannels showed considerably stronger reductions in current at positive voltages compared with Cx26 hemichannels (see also currents in Fig. S3). This robust decrease in conductance at positive voltages is ascribable to a second gating mechanism present in Cxs that closes these hemichannels to a substate at positive membrane voltages (Bukauskas and Verselis, 2004). This gating mechanism exhibits a higher sensitivity to voltage in Cx30 than in Cx26.

### Cysteine substitution at position 49 does not affect Cx26 and Cx30 GJ channel function

**Figure 9.**
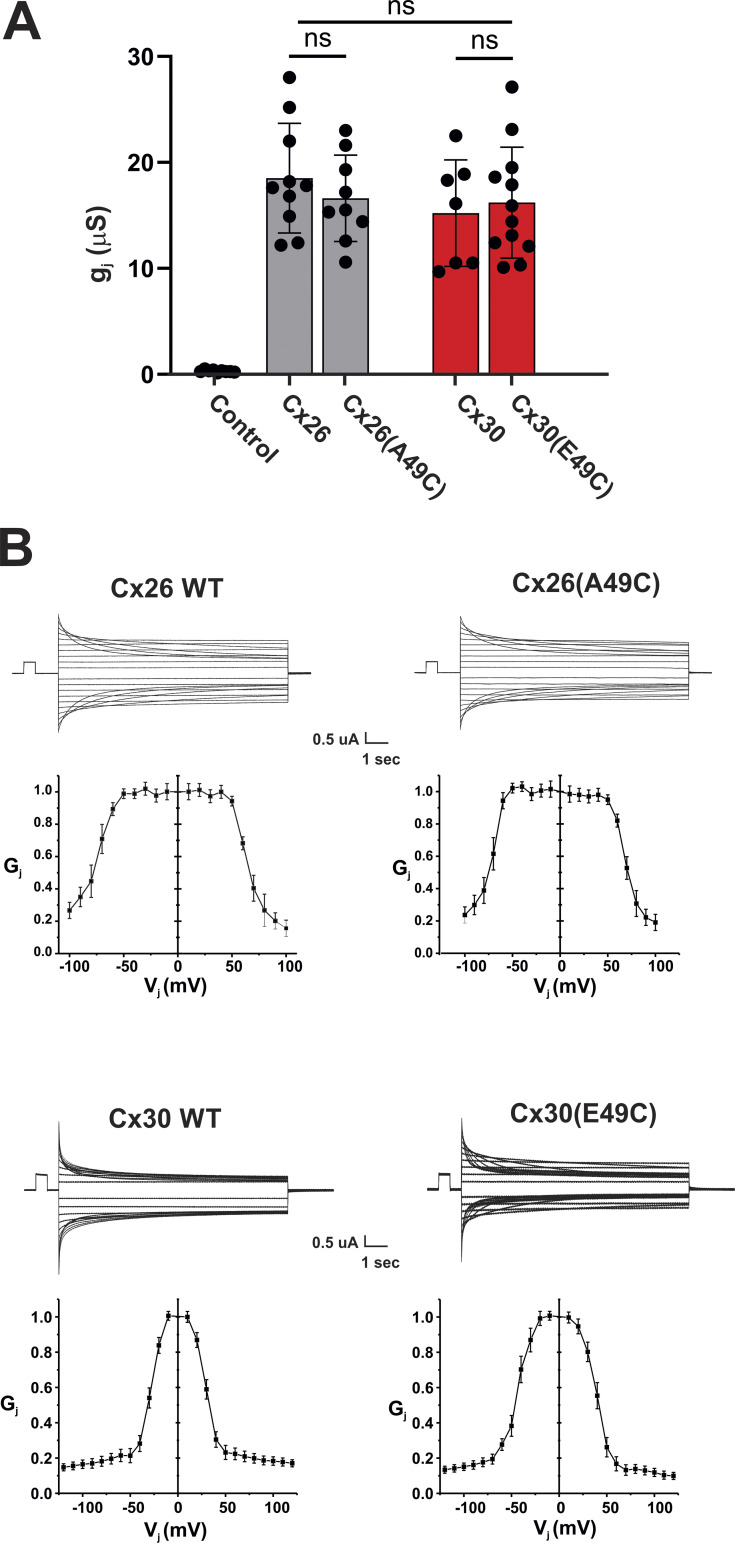
**Cx26 and Cx30 GJ currents are robust and unaffected by Cys substitution at position 49. (A)** Bar graph showing mean values for junctional conductance, gj, measured in oocyte pairs injected with WT Cx26 (*n* = 10), Cx26(A49C) (*n* = 9), WT Cx30 (*n* = 7) and Cx30(E49C) (*n* = 13). Control oocytes were injected with water (*n* = 5). Conductances were measured from currents in response to ±10 mV steps from a common holding potential of −40 mV. Filled circles represent individual measurements in different cell pairs. Bars represent mean ± SD. Significance among groups was tested using a one-way ANOVA and post-hoc Tukey’s adjusted for pairwise comparison. ns denotes that there were no significant differences between the junctional conductance measurements. P values for Cx26 versus Cx30 (0.5357); Cx26 versus Cx26(A49C) (0.8365) and Cx30 versus Cx30(E49C) (0.9741). **(B)** Mean steady-state G_j_-V_j_ relationships obtained from subsets of the cell pairs examined as in A. Values represent mean ± SE. Each G_j_ represents g_j_ normalized to the maximum at V_j_ = 0 in each cell pair. V_j_ steps between ±100 mV were applied in 10 mV increments. Records were taken from oocytes that were paired for 24–36 h. Representative examples of junctional currents are shown for WT Cx26 and WT Cx30 and the corresponding and Cys-substituted mutants at position 49. Control (uninjected) (*n* = 9); Cx26 (*n* = 10); Cx30 *n* = 7); Cx26(A49C) (*n* = 9); Cx30(E49C) (*n* = 12).

Unlike Cx30 hemichannels, Cx30 GJ function was robust much like that of Cx26 GJs indicating that compromised hemichannel function influenced by the charge pair at positions 49 and 50 does extend to GJ channels. Also, GJ channel functionality was not affected by Cys substitution at position 49. Both Cx26 and Cx30 GJ channels carrying the A49C substitution were functional without the addition of DTT or TPEN. Mean levels of junctional currents 24 h after pairing were comparable (Fig. 9 A). Moreover, junctional currents and steady-state g_j_-V_j_ relationships for Cys substituted variants were comparable with their WT counterparts (Fig. 9 B). These data suggest that conformational changes associated with docking occur and alter the structure of the E1 domain in a way that changes the nature of the local interactions involving positions 49 and 50, and perhaps other positions.

### Ca^2+^ waves and the distribution of Cx26 and Cx30 in the developing cochlea

**Figure 10.**
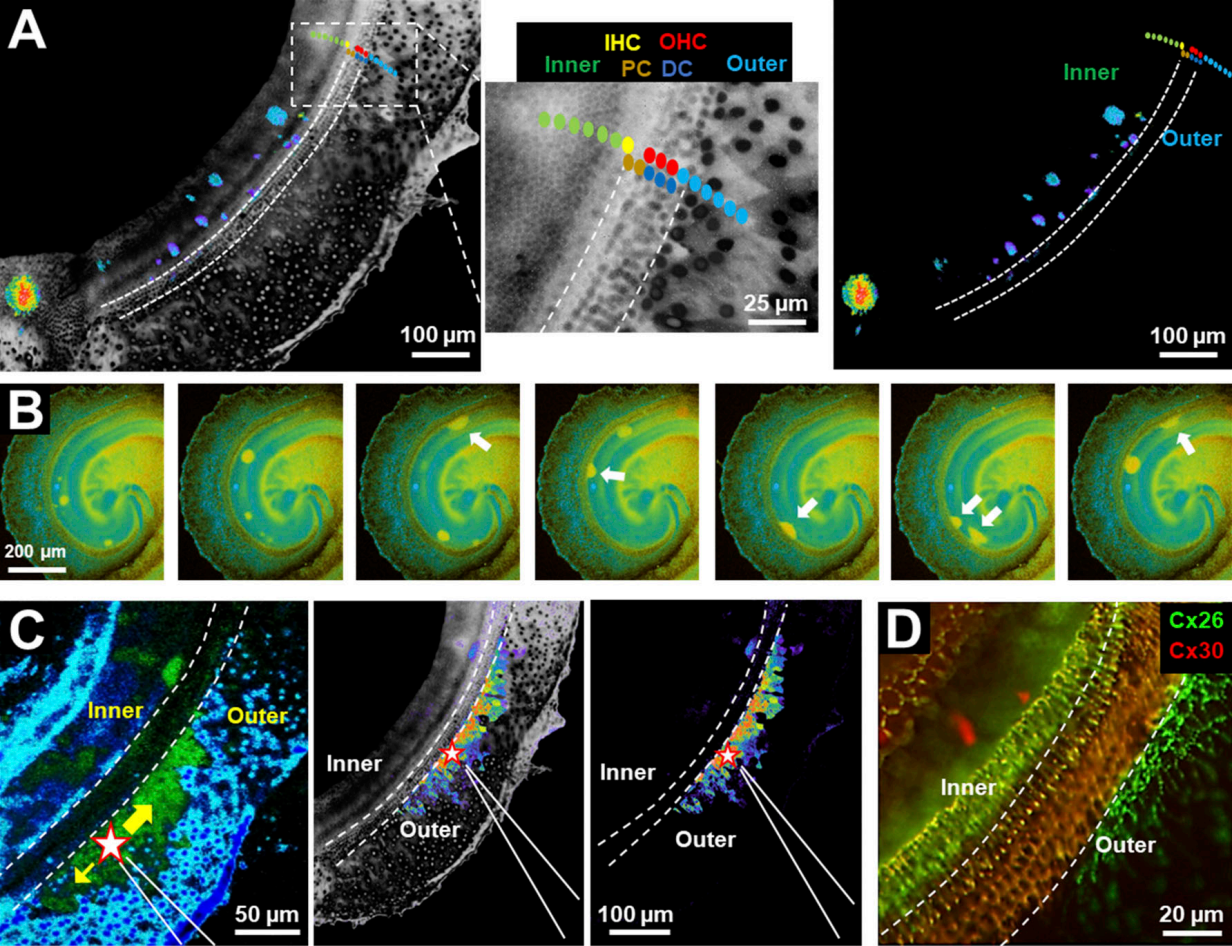
**The hair cell region in postnatal cochlear explants presents a barrier to spontaneous and induced Ca**^**2+**^ **waves and shows higher Cx30 expression.** Experiments shown were performed on a mouse explant culture extracted at P7 and recorded 2 days later (P7+2). The area examined was in the medial/apical region of cochlea. **(A)** An integrated intensity projection of the spontaneous Ca^2+^ waves (left panel) shows activity confined to the inner supporting cells. Integration was performed over 84 frames (168 s total elapsed time). An expanded view (inset) of the boxed segment illustrates the approximate positioning of the various cell types: the inner supporting cells (Inner), the inner hair cell (IHC), the pilar cells (PC), the Deiter’s cells (DC), the outer hair cells (OHCs), and the outer supporting cells (Outer). The right panel shows an intensity projection of the same data as in A with the background image masked to emphasize the Ca^2+^ activity and its restricted pattern within the inner supporting cell region. The dotted lines demarcate the region from the border with the IHCs to the outer border with the OHCs. **(B)** Individual examples of spontaneous Ca^2+^ waves in another cochlear explant. Images represent snapshots of Ca^2+^ waves close to their maximal extent of spread. Spherical waves of varying size resulted when they originated within the inner supporting cell region away from the border with the IHCs. Ca^2+^ waves that originated at the IHC cell border were dome-shaped due to restricted spread away from the hair cell region. **(C)** Example of a Ca^2+^ wave induced in the outer supporting cell region of the same explant as in A with a puff of ATP (40 µM) from a positioned pipette. The star indicates the initiation site of the Ca^2+^ wave near the pipette tip. The dotted lines demarcate the region from the border with the IHCs to the outer border with the OHCs. Intensity projections with and without the cochlear background image illustrate the preferential travel of the Ca^2+^ wave within the outer supporting cells. Integration was performed over 30 frames (60 s elapsed time). **(D)** Antibody-stained image of a similarly aged explant showing Cx26 and Cx30 expression with relatively high levels of Cx30 in the hair cell region that tended to act as a barrier to the propagation of Ca^2+^ waves between inner and outer supporting cell regions.

Spontaneous and ATP-evoked Ca^2+^ waves have been shown to be prevalent in the developing cochlea and appear to influence the development and acquisition of a normal hearing phenotype (Mammano and Bortolozzi, 2018). Cxs play a central role in the generation and propagation of these Ca^2+^ waves, both through the release of ATP by hemichannels and the GJ-mediated intercellular transfer of signaling molecules, such as IP_3_ (Anselmi et al., 2008; Ceriani et al., 2016; Decrock et al., 2012; Majumder et al., 2010; Tritsch et al., 2007). Given the differential functionality, we found for Cx26 and Cx30 hemichannels, we examined whether the relative expression patterns for Cx26 and Cx30 in developing mouse cochlea impacted the observed patterns of Ca^2+^ activity. Because Cx30 hemichannels function poorly, support for Ca^2+^ wave propagation could be diminished where Cx30 expression predominates. To assess this possibility, we utilized in vitro explant cultures of the organ of Corti extracted from postnatal mouse pups and loaded them with the ratiometric Ca^2+^-sensitive dye Fura-2 AM. In the postnatal cochlea, prior to the onset of hearing at ∼P12, spontaneous Ca^2+^ waves have been shown to be common within the inner supporting cells that make up the greater epithelial ridge, which at early stages of postnatal development encompasses Kolliker’s organ characterized by immature, columnar-shaped supporting cells (Majumder et al., 2010; Mammano and Bortolozzi, 2018). Fig. 10 illustrates the Ca^2+^ dynamics that occur in an explant culture isolated from a P7 mouse recorded after 2 days in culture (P7+2). Recordings were done in low Ca^2+^ (0 mM added), a condition that should promote both Cx26, and to a lesser extent, Cx30 hemichannel opening; physiologically, endolymphatic Ca^2+^ levels are very low (Wangemann, 2006). Integrated intensity projections of spontaneous Ca^2+^ fluctuations over time (Fig. 10 A) illustrate that the Ca^2+^ activity is largely confined to the inner supporting cell region in agreement with previous reports (Majumder et al., 2010; Rodriguez et al., 2012). The spontaneous activity was suppressed in the presence of 2 mM extracellular Ca^2+^ and robustly enhanced in low Ca^2+^ when paired with K^+^-induced depolarization; the latter further increases hemichannel opening in low Ca^2+^ through voltage-dependent activation (Fig. S6). These data are consistent with Cx hemichannels playing a role in the observed spontaneous Ca^2+^ wave activity.

The characteristics of the spontaneous Ca^2+^ waves are illustrated in Fig. 10 B. In the representative examples shown, Ca^2+^ waves that initiated in the inner supporting cells, away from the border region containing inner (IHC) and outer (OHC) hair cells, generally spread radially for finite, but variable distances, resulting in spherically shaped Ca^2+^ transients. However, waves that initiated in the inner supporting cells close to the border with the IHCs failed to spread further into the hair cell region resulting in distorted dome-like shaped Ca^2+^ transients (arrows) due to preferential spread away from and along the border. Although the outer supporting cells showed little spontaneous Ca^2+^ activity, Ca^2+^ responses could be elicited with focal application of ATP via a pipette (40 μM; star). An example of an ATP-induced Ca^2+^ response initiated in the outer cells is shown in Fig. 10 C. The Ca^2+^ response from the region surrounding the pipette tip showed preferential travel within the confines of the outer supporting cells, better illustrated with integrated intensity projections of the induced Ca^2+^ activity recorded as the wave propagated. Thus, the hair cell region, which contains developing Pillar and Deiter’s supporting cells, acts as an impediment to the spread of Ca^2+^ waves between inner and outer compartments. Antibody staining of similarly aged cochlear explants showed widespread Cx26 and Cx30 co-expression throughout the supporting cells, as reported in a number of studies (Forge et al., 2013; Jagger and Forge, 2006; Majumder et al., 2010; Sun et al., 2005; Zhao and Yu, 2006). However, within the hair cell region that acted as an impediment to the spread of Ca^2+^ waves, there was a relatively high level of Cx30 expression supporting the view that reduced Cx30 hemichannel function shapes the patterns of wave propagation observed in the developing cochlea (Fig. 10 D).

## Discussion

Undocked hemichannels represent a Cx channel configuration that can play important roles in tissue function separate from GJ channels by mediating the exit/entry of ions and larger signaling molecules across the plasma membrane. In our examination of Cx26 and Cx30, two closely related Cxs that share widespread expression in the cochlear epithelial cell networks, we found different capacities for hemichannel function, particularly in the presence of extracellular Ca^2+^. We also found that an Ala/Glu difference at position 49 in the proximal portion of the E1 domain in Cx26, designated as a parahelix (Kwon et al., 2011, 2012), could account for this differential capacity.

Although we did not make a direct assessment of surface Cx expression in our studies in *Xenopus* oocytes, our data compellingly suggest that the relatively low levels of functional Cx30 hemichannel currents are not caused by a deficiency in Cx30 protein expression or membrane insertion, but rather by a reduced functional capacity. This conclusion was based on a number of criteria, including injecting the same concentrations of Cx26 and Cx30 cRNA, observing high expression of Cx30 with fluorescent tagging despite showing low levels of functional hemichannel currents, observing high levels of Cx30-mediated GJ currents under the same conditions, and showing that Cx30 contributed to the expression of large, Ca^2+^-sensitive hemichannel currents when co-expressed with Cx26(D50N/A), KID syndrome mutants in which position 50 is neutralized. This conclusion is consistent with observations made by Abbott et al. (2023), who reported that currents mediated by Cx26/Cx30 heteromeric hemichannels, but not Cx30 homomeric hemichannels, could be readily detected in solutions containing Ca^2+^.

Investigations of Cx30 that sought to assess the potential roles of Cx hemichannels in astrocytes reported substantial Cx30 hemichannel currents expressed in *Xenopus* oocytes (Hansen et al., 2014a, 2014b; Nielsen et al., 2017, 2019). Notably, these currents were observed in solutions devoid of divalent cations, Ca^2+^ and Mg^2+^. We also reliably observed Cx30 currents in the absence of added Ca^2+^ (Fig. S3), but under similar conditions, Cx26 produced considerably larger currents, so much so that it led to an inability to maintain voltage clamp with the imposition of moderate voltage steps. Therefore, although the ability of Cx30 hemichannels to function is not in dispute, we view that Cx30 hemichannels have a lower intrinsic capacity for function than Cx26 hemichannels over a broad range of divalent cation concentrations.

### The Ala/Glu difference at position 49 and Cx26 and Cx30 hemichannel functionality

A49 in Cx26 resides a region designated as a parahelix, residues E42—F51, in a refined, equilibrated structure derived from computational studies that filled in missing coordinates in the original Cx26 GJ channel crystal structure and applied all-atom molecular dynamics simulations (Kwon et al., 2011, 2012). The refined structure retained the same gross features as the crystal structure but contained some changes in helix packing, positioning of residues at the interfaces of the transmembrane domains and alterations in the contour of the aqueous pore. However, relevant to our studies here, the putative pore-lining E1 residues, A49, G45, D46, and D50, all maintained a high probability of pore exposure in the parahelical conformation (Kwon et al., 2012).

Applying SCAM using MTS reagents, we confirmed that position 49 is pore-lining in functionally active Cx26 hemichannels, joining two other parahelical positions, 45 and 50, confirmed in the same manner. However, the effects of MTS modification of A49C differed from the effects at these other positions. In G46C and D50C hemichannels, MTSET and MTSES produced rapid, sustained changes in current that were opposite in sign; MTSET produced a reduction and MTSES an increase as a result of effects on open channel conductance (Sánchez et al., 2010; Sanchez et al., 2013). In A49C hemichannels, both MTS reagents produced robust reductions in current, but it occurred in two phases. A transient increase in current preceded the robust decrease for MTSES and a rapid decrease preceded a slower decrease for MTSET. Remarkably, neutralization of D50 dramatically changed these effects such that MTSES now produced a rapid, sustained increase in current and MTSET a rapid sustained decrease, resembling the effects in G45C and D50C hemichannels. We conclude that MTS modification of A49C results in two effects, a rapid one reflecting a change in the open channel conductance and a slower one that leads to robust hemichannel inhibition. Neutralization of D50 abolishes the second, slower effect leaving only rapid effects that are opposite in sign for MTSET and MTSES, consistent with changes expected by introducing oppositely charged side chains in the permeation pathway.

### Local pore interactions in E1 and hemichannel function

Mechanistically, the slower, inhibitory effects of MTS modification in A49C hemichannels could occur by promoting closure or by occluding or blocking the pore. Block would have to occur following the initial rapid effects that we ascribe to altered conduction through the open pore and thus, in essence, would represent a subsequent conformational change following MTS modification. Shifts in hemichannel activation positive along the voltage axis associated with MTS-induced reductions in current support, a gating rather than a blocking mechanism. Positive shifts in activation were also observed with A49K and A49E charge substitutions. These results together with the loss of the inhibitory effect upon neutralization of D50 suggest that electrostatic interactions between these adjacent pore-lining residues at positions 49 and 50 strongly influence hemichannel open/closed equilibria.

The consequence of the shifts in the G-V relations would be to substantially reduce hemichannel currents at typical resting potentials, even with low levels of extracellular Ca^2+^. Given that the introduction of charge of either sign at position 49, whether by charge substitution or MTS modification, produced similar shifts, both attractive and repulsive electrostatic interactions would appear to promote similar effects. Alternatively, position 49 may be particularly susceptible to substitutions so that different charges and/or chemical compositions and/or volumes of the side chains would all lead to a similar phenotype. This possibility was not systematically explored, but MTSES, which imposes additional steric effects due to a larger-sized side chain, did produce a larger shift than an A49E substitution. In any case, the neutralization of D50 clearly impacted the effects of all the A49 substitutions and modifications imposed by reducing the tendency toward hemichannel closure, particularly at hyperpolarized potentials.

### Cx channel gating mechanisms are linked to pore-lining residues

Voltage-dependent gating in Cx channels was originally described in studies of GJs between electrically coupled amphibian blastomeres and showed a unique response to the voltage difference between the cells, the transjunctional voltage, V_j_ rather than their transmembrane voltages (Harris et al., 1981; Spray et al., 1981). Sensitivity to V_j_ was explained by positioning of voltage-sensing residues within the aqueous pore where they experience the electric field between the cells rather than across the plasma membrane (Bukauskas and Verselis, 2004; Harris et al., 1981). Mechanistically, gating turns out to be a hemichannel property so that closure of a GJ channel occurs by closing one or the other hemichannel depending on the polarity of the transjunctional voltage (reviewed in Bukauskas and Verselis, 2004). Thus, the same gating mechanisms apply when considering GJ channels and undocked hemichannels.

Undocked hemichannels represent a simpler configuration to examine gating and clearly reveal the existence of two distinct voltage-dependent gating mechanisms (Trexler et al., 1996). The mechanism by which hemichannels activate in the plasma membrane upon membrane depolarization was termed loop gating, as it was ascribed to movements of the extracellular loops (Trexler et al., 1996). This mechanism acts together with extracellular Ca^2+^ to play a critical role in modulating hemichannel activity and preventing excessive exit/entry of molecules that could compromise cell integrity. Although biophysical, molecular, and structural studies have not yet provided a consensus regarding the site of Ca^2+^ binding or the mechanism of action in molecular detail, evidence points to Ca^2+^ acting as a modulator of voltage-dependent gating, in essence, stabilizing the closed conformation of the loop gating mechanism (Pinto et al., 2017; Verselis and Srinivas, 2008).

A connection between loop gating and the first extracellular E1 loop was reported in studies of Cx50 and Cx32(43E1) hemichannels; the latter is a chimera in which the E1 domain of Cx43 was substituted into Cx32 (Tang et al., 2009; Verselis et al., 2009). Substitutions and modifications of E1 residues were found to selectively modulate loop gating. Here, we similarly find that the nature of the residues at positions 49 and 50 in Cx26 and Cx30 appear to impact the loop gating mechanism, affirming the strong connection between the E1 region of the pore and Cx hemichannel function.

In Cx26 and Cx30, the other gating mechanism operates inside positive voltages and reduces conductance partially by virtue of gating to a subconducting state rather than a fully closed state. This mechanism has been proposed to act by narrowing the cytoplasmic entrance of the hemichannel through movements of the N-terminal domain (Ri et al., 1999). Although peripheral to this study, we note that Cx30 hemichannels exhibit a higher sensitivity to depolarizing voltages compared with Cx26 hemichannels.

### Functional differences between Cx26 and Cx30 hemichannels

The natural occurrence of an E49–D50 charge pair in Cx30 as an underlying factor for the inherently lower level of Cx30 hemichannel function is supported by neutralization of either E49 or D50, which led to robust hemichannel currents. Likewise, the imposition of a charge pair in Cx26 hemichannels led to reduced currents at hyperpolarized voltages caused, at least in part, by shifts in the G-V relationships. However, despite exhibiting low currents, WT Cx30 hemichannels did not exhibit a substantially shifted G-V relationship, although substitutions and MTS modifications at position 49 showed similar trends and shifts as in Cx26. A mechanistic explanation may lie in the complex nature of loop gating. Mathematical modeling studies suggest that voltage-dependent transitions to a closed state can proceed to a second closed state in a voltage-independent manner, provisionally called the deep-closed state characterized by exceedingly long dwell times that could contribute to the apparent poor functioning of Cx channels despite high levels of membrane expression (Kraujalis et al., 2022; Snipas et al., 2016). Certain stimuli, notably divalent cations and low pH, can drive Cx channels, both GJ channels and undocked hemichannels, into prolonged periods of closure (Kraujalis et al., 2022; Snipas et al., 2016; Trexler et al., 1999), consistent with entry into a deep-closed state. Thus, it is possible that Cx30 hemichannels, due to the presence of an E49–D50 charge pair, have a higher probability of entering into the deep-closed state, thereby keeping macroscopic currents at low levels without appreciable shifts in the G-V relationship. Given the important role of D50 in the dependence of Cx26 hemichannels on extracellular divalent cations (Kwon et al., 2012; Lopez et al., 2013; Sanchez et al., 2013), neutralization of D50 could reduce the tendency of Cx30 hemichannels to transit into a long-lived closed state, leading to enhanced currents. This possibility is not mutually exclusive with shifts in the G-V relationships that also affect the magnitude of hemichannel currents as a function of voltage.

### Conformational flexibility in E1 of Cx26 and Cx30 hemichannels

Disulfide bond formation and metal-thiolate bridging at substituted Cys residues has been used extensively to probe ion channel states and movements associated with gating (Bassetto et al., 2020; Henrion et al., 2012; Holmgren et al., 1998; Linsdell, 2015). The effects of Cys substitution at position 49 in both Cx26 and Cx30 hemichannels suggest that there is considerable local conformational flexibility inherent in this region of E1. Cys substitution essentially abolished hemichannel function irrespective of the voltage applied or the Ca^2+^ concentration used. Function could be restored, but required DTT, at least initially, indicative of disulfide bond formation. Following the application and washout of DTT, the application of TPEN alone could induce and sustain hemichannel function. Given that TPEN has no reducing capacity, but can effectively chelate transition metals, Cys-substitution at position 49 also appears capable of promoting the coordination of metals. Indeed, submicromolar concentrations of Cd^2+^ following opening produced robust inhibition of hemichannel currents, as did simply washing out TPEN consistent with the creation of a metal-binding site with an affinity high enough such that contaminant and/or endogenous levels of thiophilic metals present in the solutions and/or cells are sufficient to promote a metal-bound state. The effectiveness of TPEN alone, however, diminished over time and required DTT to re-establish function. These results suggest that disulfide bonds involving the substituted-Cys side chain at position 49 dominate in resting cells, thereby requiring DTT to promote opening. Following opening, the exposed sulfhydryl groups can participate in metal coordination prior to reforming disulfide bonds, allowing TPEN alone to induce function. The high affinity for metal binding suggests that the effects of metal coordination could be fairly rapid. Longer residence in the metal-coordinated state would appear to promote the eventual reformation of disulfide bonds thereby resetting the requirement for DTT.

The formation of disulfide bonds involving A49C residues requires that the Cα atoms come into close enough proximity, estimated to be at an average distance of ∼5.6 Å. With a pore diameter estimated to be ∼15 Å in the open state at the parahelix, considerable movements likely occur that leave the pore constricted at A49C following disulfide formation, abolishing current flow. Similarly, metal coordination could involve movements that constrict the pore as well with estimated average distances between Cα atoms somewhat farther apart, ∼6.5–8.0 Å, depending on the coordination number. Because coordination involves pore-lining residues, the coordinated metal ion within the permeation pathway could also abolish ion flux through the pore.

The constriction of the pore in the parahelical region depends, of course, on which residues are actually involved in these processes. Removal of the carboxylate at position 50 with either a D50A or a D50N substitution disrupted both disulfide bond formation and high-affinity metal coordination, which could occur by some general conformational change. However, we examined the effects of Cys-substitutions and neutralization of D50 at nearby residues, Q48 and G45. Substitutions at both these positions result in high-affinity metal coordination assayed by the application of 1 μM Cd^2+^. Moreover, Cys-substitution at Q48 behaved similarly to A49C, promoting both disulfide bond formation and metal coordination. However, unlike for A49C, neutralization of D50 did not significantly change the effects of 1 μM Cd^2+^ (Fig. S7) nor did it disrupt the ability of Q48C to form disulfide bonds or change the effects of MTS modifications (Fig. S8). Thus, it is likely that the effects of neutralization of D50 involve specific interaction with A49C.

### Metal bridge formation in Cxs and the pore-lining segment of the E1 domain

Cd^2+^-thiolate bridge formation was reported in Cx50 and chimeric Cx32*43E1 hemichannels (Tang et al., 2009; Verselis et al., 2009). Cx32, the backbone Cx of the Cx32*43E1 chimera, shares high homology with Cx26 and Cx30, all of which belong to Group II or the β subgroup of the Cx gene family, whereas Cx50 is more distantly related and belongs to the Group I or α subgroup; Cx50 is most closely related to Cx46 (Cruciani and Mikalsen, 2006). In Cx32*43E1 hemichannels, Cys substitutions at a total of 16 positions throughout the putative hemichannel pore-lining domains spanning from intracellular to extracellular entrances were examined (Bargiello et al., 2018). Remarkably, metal bridge formation was confined to Cys substitutions at three parahelical E1 residues, A43, G45, and A50. Notably, missing is position 49; Cys substitution at position 49 in Cx32*43E1 was reported to abolish the hemichannel function. For Cx50, pore-lining E1 residues F43 and G46 showed high-affinity metal bridge formation. Although the data set is rather limited, the pore-lining segment in E1 appears to show a unique susceptibility for metal-bridge formation in several Cx hemichannels, suggestive of a general regional flexibility among Cxs that could function in closing and opening of the pore, even though specific positions among the Cxs that show a capacity to participate in Cd^2+^-thiolate bridging may differ.

Differences in the positions that participate in Cd^2+^-thiolate bridging among Cxs is not surprising given the growing list of Cx channel structures emerging from cryo-EM studies showing that Cx pores can differ substantially in their steric and electrostatic features, which presumably underlies the wide-ranging differences in unitary conductance and permeability characteristics documented over the years among channels formed of members of the Cx gene family (Harris, 2001; Harris, 2007; Lee H. J. et al., 2023a; 2023b; Myers et al., 2018). Even for Cx26 and Cx30, which share high sequence homology, molecular dynamics simulations of Cx30 (by homology modeling) suggest different positions of major energy barriers to ionic flux, which were suggested to be at K41 in Cx26 and E49 in Cx30 (Zonta et al., 2012). Given the unique association between the pore and gating in Cx channels, these differences may not only affect unitary conductance and permeability, but the stabilities of open and closed conformations as well through altered interactions among residues that modulate loop gating, as we find here.

### GJ channel function versus hemichannel function

Cx26(A49C) GJ channels were functional without DTT or TPEN, unlike their undocked hemichannel counterparts. The same was previously found for Cx26(Q48C) GJ channels (Sanchez et al., 2013). For A49C GJs, coupling was robust, assayed 12–24 h after pairing with levels indistinguishable from WT Cx26, and no noticeable differences in response to transjunctional voltage. Cx30-mediated GJ coupling was also robust despite the low levels of Cx30 hemichannel currents assessed in the same cells. For the Cys substituted A49C and Q48C variants, hemichannels poised to dock would be non-functional due to disulfide bond formation. This condition, however, does not interfere with GJ formation and function as robust coupling develops.

Given that docking is mediated by the extracellular loops, it is possible that perturbations in the local structure of E1 impose a mechanical strain on the disulfide bonds causing them to break (Passam and Chiu, 2019) or produce structural changes such that cytoplasmic reducing factors, such as glutathione, gain access to the sites. Once an intercellular GJ channel forms, the pore is chemically and electrically sealed from the extracellular space and thus shielded from oxidation. It is unclear whether a reducing environment in the pore is needed to maintain A49C GJ channels open or whether docking results in reduced flexibility that restricts the motions of these Cys-substituted positions so that they can no longer come in close enough proximity to form disulfides or bind metals with high affinity. Given that gating is a hemichannel property, docked E1 domains in GJ channels must still retain sufficient motions for gating to occur. For Cx30, in which the E49–D50 charge pair appears to be a contributing factor to dampened hemichannel function, disulfide bond formation in GJs is not at play, suggesting docking likely imposes constraints on movements in E1, thereby greatly reducing or abolishing the effect of the charge pair in Cx30 GJs that is readily manifested in undocked hemichannels. This effect may also be linked to the loss of an accessible Ca^2+^ binding site upon docking. Of note, D50N and D50A KID mutations in Cx26 also exhibit differential effects on undocked hemichannels and GJ channels, in this case abolishing GJ channel function while maintaining hemichannel function (Lee et al., 2009). Thus, residues in this region of E1 clearly can differentially affect hemichannel and GJ channel functionality.

A recent structural study of Cx43 GJ channels and hemichannels in nanodiscs showed that both the extracellular loops, E1 and E2, engage in docking and show considerable conformational changes, although resolution was insufficient to identify orientations of individual side chains (Qi et al., 2023). Taken together, these data suggest that the E1 domains likely undergo sufficient conformational changes upon docking that, indeed, can abrogate the effects of charge or other interactions manifested in undocked hemichannels. As yet, there are no high-resolution structures of the same Cx in the GJ channel and undocked hemichannel configurations to provide a definitive answer regarding structural alterations with docking.

### Differential Cx26 and Cx30 hemichannel function and implications for the cochlea

The new consideration here regarding the potential roles of Cxs in hearing relates to the impact of Cx30 expression in the cells that make up the cochlear cellular networks. In rodents, supporting cells in the organ of Corti can show substantial co-expression or preferential expression of Cx26 or Cx30 (Forge et al., 2013; Jagger and Forge, 2006; Majumder et al., 2010; Sun et al., 2005; Zhao and Yu, 2006). Although Cx26/Cx30 have been shown to form functional heteromers in exogenous expression systems (Naulin et al., 2020; Yum et al., 2007), their formation in cochlear supporting cells has been inferred from biochemical studies showing co-assembly (Ahmad et al., 2003; Defourny et al., 2019). In human cochlea, the situation appears to differ. Studies using super-resolution fluorescence microscopy reported that Cx26 and Cx30 were expressed in separate clusters or plaques, both in the sensory epithelium and the lateral wall (Liu et al., 2016, 2017). Thus, homomeric Cx26 and Cx30 hemichannels may be the dominant forms in humans.

Our data suggests that hemichannel activity would be dampened in cells with exclusive or higher levels of Cx30 expression. In cells where Cx30 and Cx26 segregate as homomers, Cx30 hemichannels would have less impact on mediating signaling across the plasma membrane, leaving these functions largely dependent on Cx26 hemichannels, perhaps contributing to the greater impact of losing Cx26 in sensorineural deafness. Given the proposed role of ATP release through hemichannels in the generation and propagation of spontaneous Ca^2+^ waves in the developing cochlea (Anselmi et al., 2008; Majumder et al., 2010; Mammano, 2013; Mammano and Bortolozzi, 2018), our data suggest that Ca^2+^ wave propagation, indeed, is dampened in regions with increased Cx30 expression. We noted that the differential functional capacities of Cx30 and Cx26 hemichannels we observed in an exogenous expressions system may differ somewhat in native cochlear supporting cells, perhaps through the influence of factors such as the lipid environment or soluble cytoplasmic molecules.

Although it is still unclear what roles these spontaneous Ca^2+^ waves play, the loss of Cx expression, which is accompanied by loss of Ca^2+^ waves, was shown to prevent the maturation of inner hair cells, IHCs, during a critical period of postnatal cochlear development, promoting their subsequent degeneration leading to permanent hearing impairment (Johnson et al., 2017). Cx hemichannels in the supporting cells that exhibit spontaneous Ca^2+^ activity could contribute to IHC maturation by affecting their electrical activity through alterations in the extracellular ionic environment leading to depolarization and/or by producing a purinergic response through ATP release; each can promote downstream cascades that affect maturation. GJs channels also could provide crucial signaling through the supporting cell networks. Using Cx knock-out mouse models, preservation of Cx26 expression alone was shown to be sufficient to promote normal IHC maturation, although some partial maturation was observed in the presence of Cx30 when Cx26 was absent. These data are in accordance with a reduced function for Cx30 hemichannels.

### Considerations for Cx-mediated syndromic deafness

Loss of Cx channel function, both GJ- and hemichannel-mediated, leads to non-syndromic deafness. In cases where deafness is syndromic, i.e., accompanied by various cutaneous manifestations, aberrant or gain of Cx26 hemichannel function has been proposed to underlie disease pathogenesis, particularly in KID syndrome (García et al., 2015; Gerido et al., 2007; Lee et al., 2009; Sanchez and Verselis, 2014; Stong et al., 2006; Terrinoni et al., 2010). The most common KID mutation in Cx26 is D50N, which nearly abolishes the regulation of homomeric Cx26 hemichannels by extracellular Ca^2+^ (Sanchez et al., 2013). However, as our data here suggests, the Cx26(D50N) KID mutation also could affect the function of Cx26/Cx30 heteromers in an unexpected way, which is to augment their activity as hemichannels through the loss of charge interactions between positions 49 and 50. This possibility is supported by the generation of large, highly Ca^2+^-sensitive currents when co-expressing Cx30 with Cx26(D50N) or Cx26(D50A) and indicates that the effect of D50 neutralization reaches across subunits. The impact of this type of altered hemichannel activity in the cochlea is difficult to predict, but again would likely alter the patterns of Ca^2+^ waves, potentially affecting the normal course of hair cell maturation. Complicating interpretations of the effects of the D50 KID mutation are its disruption of GJ channel function (Lee et al., 2009) and a potential effect on hemichannel permeability, given that D50 is a pore-lining position. Nonetheless, the differential functions of Cx26 and Cx30 hemichannels and the distinct impacts of the D50N substitution on the functions of Cx26 homomeric and Cx26/Cx30 heteromeric hemichannels bring new considerations for the roles of these Cxs and in mechanisms of Cx-mediated disease pathogenesis.

Overall, we show that Cx30 hemichannels exhibit a lower capacity for function compared with Cx26 hemichannels, a property that is ascribable to a single Ala/Glu difference at position 49 in the proposed parahelical region in the first extracellular loop, E1, domain. We confirmed this position to be pore-lining, and interactions with the adjacent pore-lining D50 residue can robustly affect hemichannel functionality. The effects of Cys substitution at position 49 in both Cx26 and Cx30 hemichannels suggest that there is considerable local conformational flexibility inherent in this region of E1. GJs channel function is unaffected by the same substitutions suggesting that docking may produce structural alterations, most plausibly in the extracellular loops where docking occurs. Structural effects of hemichannel docking have not been described and await high-resolution structures of Cx26 and or Cx30 in both docked and undocked configurations. Complicating matters is the possibility of heteromer formation, which can be varied in composition and, thus, produce varied perturbations in structure. However, the results of Cx26 and Cx30 co-expression, including co-expression of Cx30 with the Cx26(D50N) KID mutant show that effects on hemichannel functionality extend, at least qualitatively, to heteromeric hemichannels.

## Data Availability

Original data from the article and supplementary material are available from the corresponding author upon reasonable request.
